# Environmental and health impacts assessment of long-term naturally-weathered municipal solid waste incineration ashes deposited in soil—old burden in Bratislava city, Slovakia

**DOI:** 10.1016/j.heliyon.2023.e13605

**Published:** 2023-02-09

**Authors:** Tomáš Faragó, Veronika Špirová, Petra Blažeková, Bronislava Lalinská-Voleková, Juraj Macek, Ľubomír Jurkovič, Martina Vítková, Edgar Hiller

**Affiliations:** a Department of Geochemistry, Faculty of Natural Sciences, Comenius University in Bratislava, Ilkovičova 6, 842 15 Bratislava, Slovak Republic; b SNM-Natural History Museum, Vajanského Nábrežie 2, 810 06 Bratislava, Slovak Republic; c The Center of Environmental Services, Ltd., Kutlíkova 17, 852 50 Bratislava, Slovak Republic; d Department of Environmental Geosciences, Faculty of Environmental Sciences, Czech University of Life Sciences Prague, Kamýcká 129, 165 00, Prague – Suchdol, Czech Republic

**Keywords:** Incineration, Leaching, Low-molecular-weight organic acids, metal(loid)s, MSWI residues, Urban soil

## Abstract

Municipal solid waste incineration (MSWI) is an effective method for reducing the volume/mass of waste. However, MSWI ashes contain high concentrations of many substances, including trace metal (loid)s, that could be released into the environment and contaminate soils and groundwater. In this study, attention was focused on the site near the municipal solid waste incinerator where MSWI ashes are deposited on the surface without any control. Here, combined results (chemical and mineralogical analyses, leaching tests, speciation modelling, groundwater chemistry and human health risk assessment) are presented to assess the impact of MSWI ash on the surrounding environment. The mineralogy of ∼forty years old MSWI ash was diverse, and quartz, calcite, mullite, apatite, hematite, goethite, amorphous glasses and several Cu-bearing minerals (e.g. malachite, brochantite) were commonly detected. In general, the total concentrations of metal (loid)s in MSWI ashes were high, following the order: Zn (6731 mg/kg) > Ba (1969 mg/kg) ≈ Mn (1824 mg/kg) > Cu (1697 mg/kg) > Pb (1453 mg/kg) > Cr (247 mg/kg) > Ni (132 mg/kg) > Sb (59.4 mg/kg) > As (22.9 mg/kg) ≈ Cd (20.6 mg/kg). Cadmium, Cr, Cu, Pb, Sb and Zn exceeded the indication or even intervention criteria for industrial soils defined by the Slovak legislation. Batch leaching experiments with diluted citric and oxalic acids that simulate the leaching of chemical elements under rhizosphere conditions documented low dissolved fractions of metals (0.00–2.48%) in MSWI ash samples, showing their high geochemical stability. Non-carcinogenic and carcinogenic risks were below the threshold values of 1.0 and 1 × 10^−6^, respectively, with soil ingestion being the most important exposure route for workers. The groundwater chemistry was unaffected by deposited MSWI ashes. This study may be useful in determining the environmental risks of trace metal (loid)s in weathered MSWI ashes that are loosely deposited on the soil surface.

## Highlights

• Forty years old MSWI residues loosely deposited in urban soil were investigated.
• Despite the high degree of ash weathering, risk metal (loid) content was elevated.
• Cd, Cr, Cu, Pb, Sb and Zn exceeded the indication criteria for industrial soils.
• Leaching in simulated rhizosphere solutions indicated limited metal (loid) mobility.
• Low human health risks and high ecological risks in some places were identified.

## Graphical abstract

**Figure:**
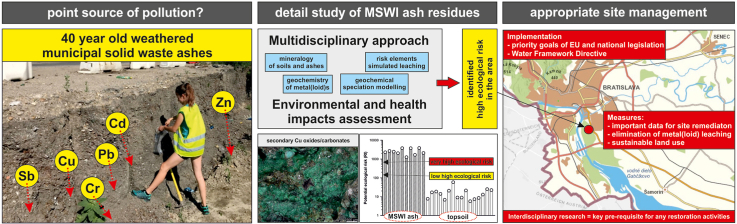

## 1 Introduction

Nowadays, a great attention is paid to the circular economy principles in different fields of industry. One of the principles of the circular economy is the waste use for secondary purposes. In the past, most of the waste of any origin was landfilled, resulting in the formation of many voluminous landfills worldwide. The total amount of municipal solid waste is continually increasing [1]. Municipal solid waste incinerators are capable to decrease remarkably the volume and mass of original waste and recover energy [[2], [3], [4]]. Bottom and fly ashes are the two main residues produced during the incineration process of municipal solid wastes [5,6]. Both original solid waste and MSWI ashes commonly contain high concentrations of trace/major metal (loid)s, such as Al, Cd, Cr, Cu, Fe, Hg, Pb, Sb and Zn, and inorganic salts (e.g. chlorides and sulphates) [[7], [8], [9], [10], [11]]. Landfilled without proper control and safety, MSWI ashes can be a source of contamination to soil, groundwater and biota with toxic chemical elements. Concentrations, chemical forms (i.e. speciation) and leaching behaviour of trace/major metal (loid)s in MSWI ashes are affected by many factors, including composition of original waste [12], incineration temperature [13], additives [6,9], waste particle size [14,15], age and weathering stage of MSWI ashes and solution pH [9,[16], [17], [18], [19], [20]]. Natural or artificial ageing of MSWI ashes is in principle a low-cost and effective method of their chemical stabilisation, which reduces the leaching of harmful chemical elements. The leaching of trace metal (loid)s in fresh MSWI ashes was studied by several authors [4,[21], [22], [23]]. Wei et al. [12], Youcai [13], Chimenos et al. [17], Polettini and Pomi [19], Su et al. [24] or Vasarevičius et al. [25] investigated the leaching behaviour of short-term naturally or artificially aged MSWI ashes. Other studies were focused on the metal (loid) leaching in long-term naturally aged MSWI ashes [17,18,20,26]. A common tendency is that the leaching of metal (loid)s in aged MSWI ashes is lower than in the fresh ones. The reduced leaching is linked with more stable mineralogical composition and different chemical properties of aged MSWI ashes compared to their fresh counterparts. Changes in mineralogical and chemical composition of fresh and aged MSWI ashes are a direct result of weathering processes that include reactions, such as carbonation, dissolution–precipitation, oxidation–reduction, sorption–desorption and complexation. Relatively fast alteration of Ca-rich phases and high leaching of inorganic salts and trace elements are typical for the initial stage of weathering. Slower Si-based glass phase alteration, extensive calcite growth and evolution of secondary mineral phases dominate in the advanced stage of weathering. In weathered MSWI ash, trace elements are mostly associated with stable secondary mineral phases [12,18,20,27]. The release of metal (loid)s from environmental solid matrices into the aqueous solution is also affected by the ubiquitous low molecular weight organic acids (LMWOAs). These organic acids are formed by the metabolism of plants and microorganisms and the decomposition of organic matter. Of the monocarboxylic LMWOAs, formic and acetic acids are the most abundant in soil solutions, while oxalic and citric acids are the most common in the di- and tricarboxylic LMWOA group, respectively [28]. They are effective complexing agents able to mobilise trace elements, and thus, generally increase their leaching in contaminated soils [[29], [30], [31]].

The aim of this study was to identify the extent of soil contamination affected by the historical uncontrolled disposal of MSWI ashes to assess the potential environmental risks for the urban area. For this purpose, the bulk chemistry and mineralogy of selected more than 40 years old MSWI ashes were determined along with the leaching behaviour of metal (loid)s when MSWI ashes interacted with dilute LMOWA solutions, specifically oxalic and citric acids. Although many aliphatic organic acids are found in soils, the most common being monocarboxylic acids such as acetic, formic and propionic acids, oxalic and citric acids were selected as representatives of di- and tri-carboxylic acids in this study. The reason for the selection was their much greater chemical reactivity (e.g. formation of complexes with metals) compared to monocarboxylic organic acids [28,[32], [33], [34]]. Last but not least aim of the study was to evaluate the impact of contaminated soils on the chemical composition of groundwater bodies in the given area. Since this is a contaminated area in the capital city (Bratislava, Slovakia), its utilization may significantly alter the geochemical characteristics in the area leading to environmental and human risks. This study may provide useful observations and conclusions on the trace metal (loid) mobility in old weathered MSWI ashes in brownfield areas and their impact on groundwater quality.

## 2 Materials and methods

### 2.1 Area description

The area is located between the municipal solid waste incinerator belonging to the OLO company and the Slovnaft refinery in south-eastern part of Bratislava city, Slovakia. The incinerator was put into operation in 1978. There was no waste separation and recycling management in the past, and therefore, hazardous materials with high concentrations of trace metal (loid)s could be included in the incinerated waste. In the 80s, the MSWI ashes (both bottom and fly ashes) were loosely deposited close to the incinerator. The landfill area has never been properly closed and isolated from the surrounding environment. The MSWI ashes were partly covered by soil and vegetation grew over the landfill during the following years. The MSWI ashes have been exposed to the atmospheric conditions and plant exudates for over forty years. Moreover, illegal municipal and construction waste depositions were found in the area during its visual inspection. Most of the ash residues was recently removed and properly landfilled. A part of the MSWI ashes could not be removed as it is situated under the existing road. Besides that, several spots of the MSWI ashes, reaching a depth of ≥1 m, are still present in the study area. A new highway is currently crossing the old landfill and buildings are under construction in the area, with the surrounding being wooded. The area is a part of an environmental burden registered as “B2 (014)/Bratislava – Ružinov – incinerator – slag dump in front of the building (SK/EZ/B2/130)” in the Register of environmental burdens. The site is built by quaternary fluvial sediments consisting mostly of well-graded gravel, gravelly sands, fine sands and flood silt. The colour of gravel varies from brown to grey and the gravel diameter ranges from 1 cm to 10 cm. The gravelly sand layers consist mostly of gravel (60–77%) and sand (10–25%). The content of clay fraction is negligible. Fine sands of fluvial–aeolian origin are commonly deposited just below the soil layers. Their thickness reaches 4 m in some parts of the area. The total area covered by the ash was about 6100 m^2^. The groundwater table is detected at a depth of 1.8 m below the surface and is not in direct contact with MSWI ashes. The aquifer is in gravelly sands and characterised by an inter-granular permeability. The hydraulic conductivity and transmissivity coefficient of the aquifer are 6.13 × 10^−3^ m/s and 4.6 × 10^−2^ m^2^/s, respectively [35]. The direction of groundwater flow is from west to east and has been highly affected by the groundwater hydraulic protection system operated by the Slovnaft refinery since 1973 [36].

### 2.2 Sample collection and processing

The location of sampling sites is shown in Figure S1 and the collected solid materials are either soils or deposited MSWI ashes that remained uncovered or only partly covered by soils (samples S1 to S10 in Figure S1). The sample at each sampling site was taken from drill cores (upper 0.2–0.3 m) in July–August 2019 and stored in plastic bags. Only samples S7 to S10 (weathered MSWI ash samples) were collected from hand–dug shallow probes, while samples S9 and S10 are from two depths: 0–0.3 m (S9a, S10a) and 0.3–0.5 m (S10a, S10b). The samples were air-dried and large pieces of foreign material, such as rocks, ceramics, glass, bricks, metallic and plant fragments were removed. Afterwards, the samples were passed through a 2 mm sieve and homogenised.

Groundwater samples were collected by short-term pumping from five 15 m-deep wells (one existing in the body of the MSWI ash landfill and four new wells designed in the direction and against the groundwater flow (Figure S1)) during April–September 2020. Basic physico-chemical parameters of groundwater samples, i.e. temperature, pH, redox potential (Eh), dissolved oxygen and electrical conductivity (EC) were determined in the field. After stabilising these parameters, the groundwater samples were filtered through a 0.45 μm filter, stored in sampling bottles and then transported to the laboratories at a temperature of 4 °C. One aliquot of the groundwater samples was acidified with ultrapure nitric acid for the determination of major and trace metal (loid)s, and the other part was left unacidified for anion analysis. All groundwater samples were stored at 4 °C until analysis. A complete chemical analysis, including major cations and anions (Al, Ca, Fe, K, Mg, Mn, Na, Cl^−^, HCO_3_^−^, NH_4_^+^, NO_3_^−^, PO_4_^3−^ and SO_4_^2−^) was done on groundwater samples from the well GW-1 (Figure S1), while groundwater samples from other four wells were measured only for trace metal (loid)s (As, Cd, Cr, Cu, Ni, Pb, Sb and Zn).

### 2.3 Chemical and mineralogical analyses

The concentrations of monitored metal (loid)s (As, Ba, Cd, Cr, Cu, Fe, Mn, Ni, Pb, Sb and Zn) in the solid samples were determined by inductively coupled plasma-atomic emission spectroscopy (ICP-AES) and hydride generation-atomic absorption spectroscopy (HG-AAS), respectively, after acid digestion of pulverised samples (<63 μm) in HNO_3_–HCl–HF mixture in accredited laboratories ALS Czech Republic, Ltd. (Prague, Czech Republic). The concentrations of other elements (i.e. Al, Ba, Ca, Cl, K, Mg, P and S) were measured only in samples subjected to leaching tests with LMWOAs solutions using the ARL Quant’X (Thermo Scientific Inc, USA) EDXRF spectrometer at the laboratory of the Slovak National Museum in Bratislava, Slovakia. The recovery of individual metal (loid)s based on repeated analysis of certified reference material (NIST 2711a – Montana II soil) ranged from 85.7% for Cr to 101% for Cd. The precision expressed as relative standard deviation (RSD) was lower than 10% for all chemical elements. The same analytical techniques (AES-ICP and HG-AAS) were used to determine major and trace metal (loid) concentrations in groundwater samples and leachates obtained by extracting selected MSWI ash samples with LMWOAs solutions (detailed below). The major anions (Cl^−^, NO_3_^−^, SO_4_^2−^) were analysed by ion chromatography (Dionex ICS-2000, Thermo Scientific).

For mineralogical analyses, sample S9a before as well as after the leaching experiment with dilute LMWOA solutions was selected to detect possible changes in mineralogical composition. This sample is representative of MSWI ash residues in soil. An amount of 1 g sieved sample and 0.25 g of 99.9% of Al_2_O_3_ powder were weighed into the grinding jar containing agate cylinders. Then, 4 mL of denatured ethanol were added. The jar was closed, shaken by hand, and then placed in a special mill (McCrone Micronising Mill) where the sample was ground for 5 min. Afterwards, the sample was transferred on a watch glass and the jar was thoroughly rinsed with denatured alcohol. The watch glass was placed in a dryer for 24 h. Mineralogical composition of the treated samples was analysed by X-ray diffraction (XRD) and Raman spectroscopy. Powder XRD analyses were performed using Philips PW 1710 diffractometer (Cu radiation with graphite monochromator at 20 mA and 40 kV, scanning range 4–65° 2Θ). The step size was 0.02° 2 Θ and exposition time 2 s/step. The results were interpreted based on the Rietveld refinement using the X'pert Highscore Plus 2.0.1 software (PANalytical B·V.). The Raman spectra of the samples were measured using Thermo Scientific DXR3xi Raman Imaging Microscope (Thermo Fisher Scientific, USA; laboratory of the Slovak National Museum in Bratislava, Slovakia). Excitation lasers wavelengths of 780 nm and 532 nm, 50 × objective, a 25 μm confocal pinhole, and an EMCCD detector were used in the study. Individual spectra were acquired as follows: mascagnite at a laser power of 38.1 mW/0.5 s (800 scans for a cycle), apatite – 34.1 mW/7.0 s (800 × ), jarosite – 0.8 mW/0.02 s (800 × ), calcite – 28.9 mW/0.125 s (500 × ), albite – 37.4 mW/0.1667 s (600 × ), brochantite – 14.9 mW/4.0 s (400 × ), malachite – 7.8 mW/0.04 s (250 × ), aurichalcite – 23.5 mW/0.1 s (250 × ), phlogopite – 11.5 mW/0.1 s (120 × ), rutile – 17.2 mW/0.1429 s (200 × ), goethite – 1.2 mW/2 s (350 × ), lazurite – 7.0 mW/0.0071 s (18 × ), hematite – 37.7 mW/0.04 s (800 × ), posnjakite – 10.1 mW/0.1667 s (180 × ) and quartz – 4.7 mW/5 s (25 × ). The processing of spectra, including fitting by Voigt functions, was carried out using the Thermo Fisher Scientific OMNIC v. 9.11 software package. Photographic documentation of micro- and macro-particles was performed by digital microscope Leica DVM6.

### 2.4 Leaching experiments

Aqueous solutions of citric and oxalic acids at two concentration levels (0.1 and 1.0 mM) were used to simulate the static leaching of metal (loid)s and anions from six MSWI ash samples with high total concentrations of metal (loid)s (S7, S8, S9a, b and S10a, b) under rhizosphere conditions. The reason was that the plant cover and soil microorganisms represent a continuous source of many organic acids, which significantly affect the adsorption and leaching behaviour of chemical elements in soils and other solid materials [[37], [38], [39], [40], [41]]. The initial pH values were 5.45 and 3.50 for 0.1 and 1.0 mM citric acid solutions, and 4.90 and 3.20 for 0.1 and 1.0 mM oxalic acid solutions, respectively. The selected concentrations of LMWOAs correspond to the range of natural concentrations found in soils [28,32,34,42,43], and are also commonly encountered in most previous studies [33,37,40,41]. A duplicated amount of 8 g sieved solid material in PE flasks (100 mL) was poured with 80 mL of LMWOA solution. The flasks were tightly closed and shaken on end-over-end shaker for 24 h at 22 °C, then centrifuged at 7000 rpm for 10 min and vacuum filtered through a cellulose nitrate membrane filter with pore size of 0.45 μm. The final pH and electrical conductivity (EC) were measured in the leachates and then stored in a refrigerator at 4 °C prior to analysis.

### 2.5 Data analyses

One of the tasks of the study was to determine the concentrations of several potentially toxic metal (loid)s in soils strongly affected by industrial activity, i.e. the enrichment of soils with these metal (loid)s. For this purpose, the enrichment factor (EF) was used, which was calculated according to the equation [44]:

(1) $$EF=\frac{{\left(C_{i}/Fe\right)}_{soil}}{{\left(B_{i}/Fe\right)}_{background}}$$

where *C*_i_ is the measured metal (loid) concentration in the soil (mg/kg) and *B*_i_ is the background metal (loid) concentration (mg/kg). Iron (Fe) was taken as the reference element [45] and metal (loid) concentrations in local soils taken from the nearby area where MSWI ash was not deposited were used as background concentrations (Table S1). According to EF values, soil enrichment with metal (loid)s is divided into the following classes [46]: EF < 2 = deficiency to minimal enrichment; 2≤ EF < 5 = moderate enrichment; 5≤ EF < 20 = significant enrichment; 20≤ EF < 40 = very high enrichment; EF ≥ 40 = extremely high enrichment.

Concentrations of target metal (loid)s in soils and MSWI ashes were compared to their indication (ID) and intervention (IT) values for industrial soils, which define soil contamination at the level of further monitoring and necessity of remediation, respectively. The values of ID and IT for individual metal (loid)s are shown in Table S1.

Soil contamination can also have a negative impact on the ecosystem of the study area. For this reason, the ecological risk was calculated according to the equation [47]:

(2) $$RI=\sum_{i=1}^{n}E_{r}^{i}$$

(3) $$E_{r}^{i}=T_{r}^{i}\times PI$$

where RI is the index of potential ecological risk, *E*_r_^i^ is the single index of the ecological risk factor, *T*_r_^i^ is the toxicity coefficient of the given metal (loid) (Table S1), PI represents the single index of contamination defined as the ratio of the measured concentration of the metal (loid) in the soil (*C*_i_) to its background concentration (*B*_i_) and *n* is the number of chemical elements included in the RI calculation. Based on RI values, ecological risk is classified into the following groups [48]: RI < 150 = low ecological risk; 150≤ RI < 300 = moderate ecological risk; 300≤ RI < 600 = considerable ecological risk; RI ≥ 600 = very high ecological risk.

Non-carcinogenic and carcinogenic health risks were calculated for intake of trace metal (loid)s through three exposure routes: (i) soil ingestion, (ii) inhalation of soil particles and (iii) dermal contact. The intended recipient of the exposure was a construction worker who worked in the area for approximately 2 years. Details of the human health risk assessment are given in Supplementary Text S1 and the values of the quantities used in the equations are in Tables S2 and S3. The upper confidence limit of trace metal (loid) concentration in soil (95% UCL) was used in the human health risk assessment and calculated by the ProUCL program, Version 5.2 [49].

Pairwise relationships among variables (metal (loid)s) were examined using Spearman's correlation analysis. The non-parametric correlation analysis was preferred due to the strongly non-normal distribution of the concentrations of individual metal (loid)s in the soils as shown by the Shapiro-Wilk test. One-way analysis of variance (ANOVA) was used to test the differences in extracted metal (loid) concentrations between the four LMWOA treatments, i.e. citric and oxalic acids at two concentration levels of 1 mM and 0.1 mM.

The geochemical program Visual MINTEQ 3 was applied to explain the speciation of metal (loid)s in the leachates in the presence of LMWOAs and the degree of leachate saturation with respect to potential solubility-controlling phases [50].

## 3 Results and discussion

### 3.1 Occurrence and enrichment of metal (loid)s in soils

**Fig. 1:**
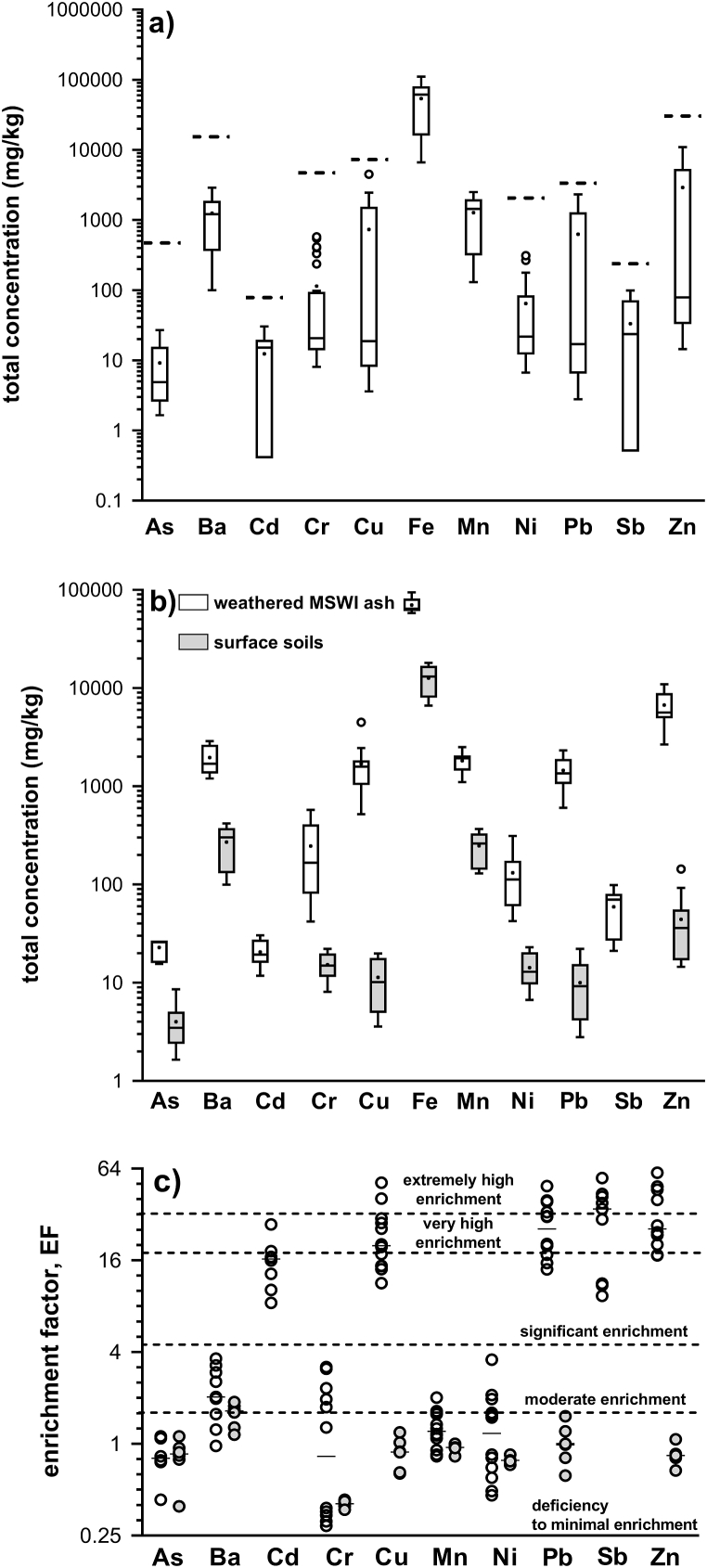
**a)** Total metal (loid) concentrations in all samples (soils + MSWI ashes, *N* = 26). The dashed horizontal line represents the intervention criteria value of the given metal (loid) (IT) for soils of industrial use, **b)** comparison of total metal (loid) concentrations between soils and historical MSWI ash residues and **c)** estimated enrichment factors (EFs) for studied metal (loid)s in soils (grey circles) and MSWI ash residues (white circles). Data in figures a) and b) are shown as Tukey box-whisker plots. The box shows the median (horizontal line inside the box) and the first and third quartiles (lower and upper horizontal lines, respectively). The length of the upper whisker is the largest value that is no greater than the third quartile + (1.5 × interquartile range). The length of the lower whisker is the smallest value that is within 1.5 × interquartile range below the first quartile. White circles are outliers. Data in figure c) for Cd and Sb in soils are not shown because total Cd and Sb concentrations were below their respective detection limits of <0.4 mg/kg and <0.5 mg/kg, respectively.

The total concentrations of each metal (loid) in the topsoil were widely variable as shown in the box plots (Fig. 1a). However, this concentration variability greatly decreased when the samples were divided into soils and those containing mostly or only MSWI ash (Fig. 1b), indicating that the high metal (loid) concentrations are associated only with the presence of incinerated wastes. The concentrations of trace metal (loid)s in MSWI ashes were in the range of those in other MSWI ashes of different ages [12,18,20,[51], [52], [53]] but higher than e.g. in fresh MSWI ashes from India [54], Vietnam [55] and China [56]. However, a high variability of their concentrations in different incinerated bottom ashes (Table S4) is evident because the resulting chemical composition of MSWI ashes is determined by the composition of incinerated waste, incineration technology and conditions, e.g. temperature, flue gas composition, residence time and humidity [56,57].

Most correlations between pairs of metal (loid)s in soils were highly significant (Fig. S2), reflecting their common source represented by buried MSWI ashes. Cadmium, Cu, Pb, Sb and Zn showed significant up to extremely high enrichment in MSWI ash residues from ten sampling sites, while soils from the remaining sites fell into the category of deficiency to minimal enrichment (Fig. 1c). As explained above, the high enrichment with Cd, Cu, Pb, Sb and Zn was related to the occurrence of MSWI ash layers. At other sampling sites, total concentrations of trace metal (loid)s were within or lower than their background values. Other trace metals, i.e. As, Cr, Mn and Ni at almost all sampling sites corresponded to deficiency or minimal enrichment, while moderate enrichment was observed for Ba, Cr and Ni in MSWI ash residues (Fig. 1c). The exceedance of ID or IT concentrations of trace metal (loid)s for industrial land use is illustrated in the form of spatial distribution maps in Figure S3. Indication and IT values exceeded Ba, Cd, Cu, Pb, Sb and Zn, total concentrations of other metal (loid)s, As, Cr and Ni, were below ID values, with the exception of one sampling site. Therefore, distribution maps for these metal (loid)s were not constructed. As shown in the spatial distribution maps (Figure S3), concentrations above the ID and IT values of all six metal (loid)s were present in the same part of the study area due to the surface occurrence of deposited MSWI ashes. This is an evidence that contamination of surface soils is only associated with historically deposited MSWI ashes, and current combustion technology with high contaminant emission control efficiency in the municipal solid waste incinerator does not produce significant emissions of trace metal (loid)s that would lead to their increased concentrations in soils in the immediate vicinity around the incinerator. Although some studies admit that municipal solid waste incineration is a source of trace chemical elements to soils [58], however, soil contamination around MSW incinerators is often the result of additional anthropogenic sources [[59], [60], [61]].

### 3.2 Mineralogy of weathered MSWI ash

**Fig. 2:**
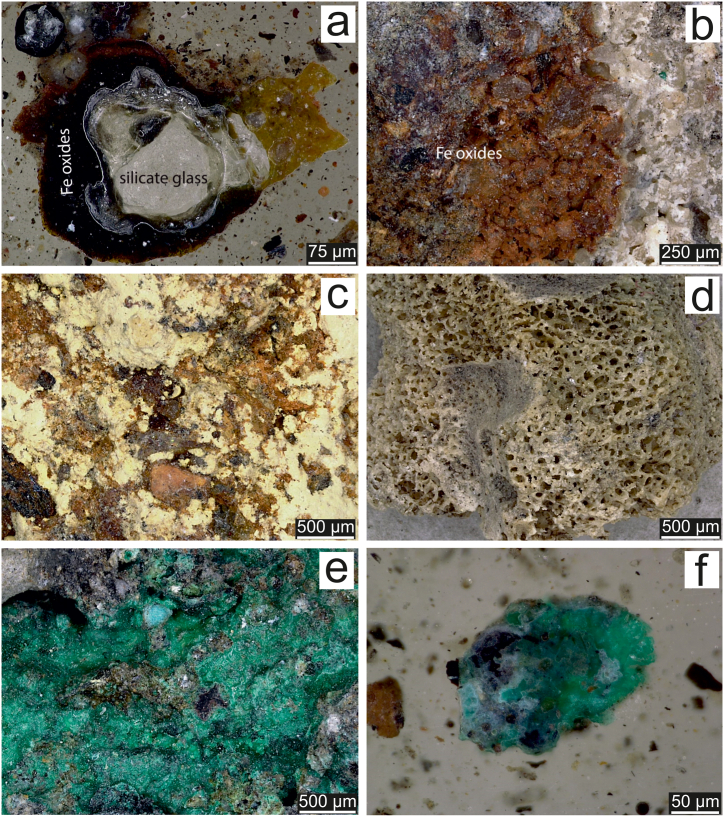
Photomicrographs of selected mineral phases in a representative sample of historical MSWI ash residues: **a)** silicate glass with a rim formed by Fe (hydr)oxides, **b)** quartz grains covered by Fe (hydr)oxides, **c)** jarosite, **d)** bone fragment, **e)** mixture of secondary Cu oxides/carbonates and **f)** grain of brochantite.

**Fig. 3:**
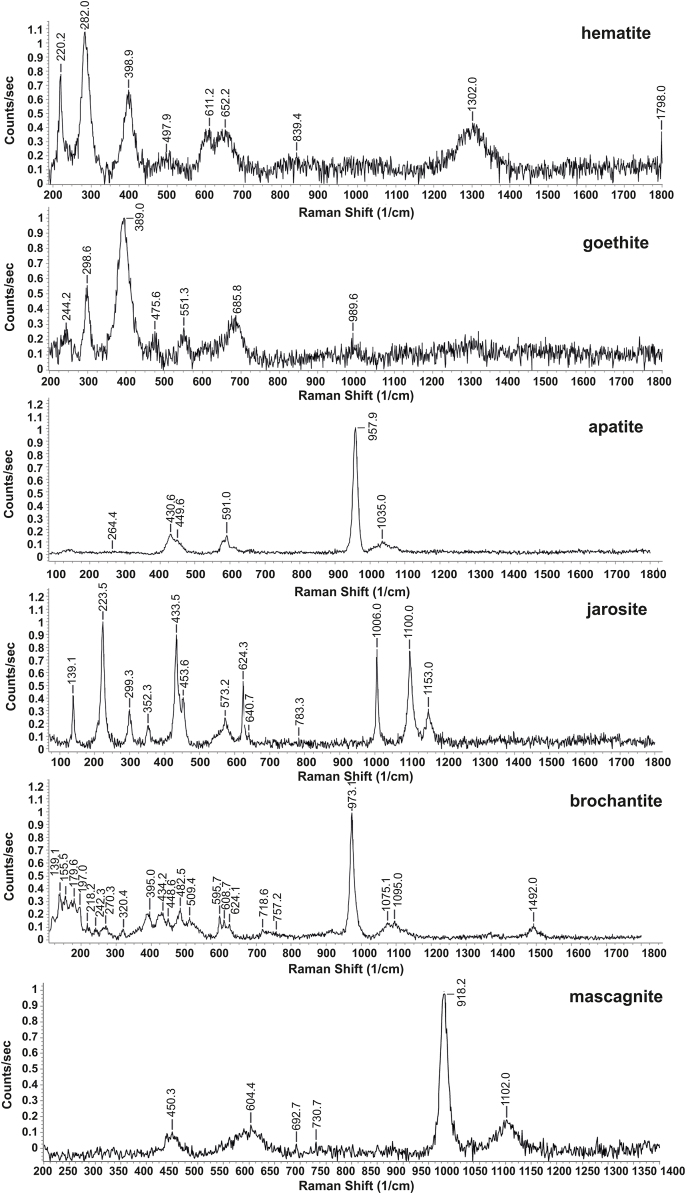
Selected Raman spectra of minerals from the MSWI ash residue (sample S9a).

The sample S9a representing weathered MSWI ash was subjected to mineralogical study. The bottom ash exposed to the atmospheric conditions for more than 40 years consisted of a variety of minerals. The most abundant minerals were quartz, maghemite, magnetite, apatite, calcite, amorphous phases (glasses and Fe (hydr)oxides) and mullite (Table 1). Melilites, hematite and other mineral phases were detected in lower proportions. Several other minerals, such as goethite, jarosite, rutile or phlogopite were identified by Raman spectroscopy (Table 1). Refractory minerals are not susceptible to weathering, therefore, they are commonly present in both fresh and aged MSWI ashes. Quartz is one of the most ubiquitous minerals in MSWI ashes and originates from incinerated municipal waste, crystallises at temperatures around 850 °C, and can even be formed by the transformation of dissolved silica during ageing [6,62,63]. Mullite is a product of thermal decomposition of clay minerals and other aluminosilicates at around 980 °C [64]. Refractory minerals are resistant to high temperatures, and therefore, usually they do not incorporate trace elements during incineration [[12], [13], [14]]. Trace elements in fresh MSWI ashes are mostly associated with primary crystalline and amorphous phases, such as melilites, pseudowollastonite, spinels and metal inclusions encapsulated in the melt glass [12,13]. Partial dissolution and mechanical damages of amorphous glass phases can lead to release of the entrapped trace elements. As the weathering proceeds, the fractures on melt glass are often covered by the layers of secondary precipitates and major, minor and trace elements are entrapped by these neo-formed mineral phases. Secondary mineral phases are usually formed by Ca, Si, Fe and Al hydrates [20], while Zevenbergen et al. [65] indicated the formation of imogolite and allophane on the surfaces of glasses in aged MSWI ash. Layers of secondary Fe (hydr)oxides were observed on quartz crystals and weathered glasses (Fig. 2a and b) but clay minerals were not identified. The Raman spectroscopy confirmed the occurrence of hematite, goethite and magnetite in the sample (Fig. 3, Table 1). Iron–bearing (hydr)oxides are found in MSWI ashes as primary or secondary mineral phases at all stages of weathering but in various proportions [51]. Saffarzadeh et al. [20] reported the occurrence of magnetite, lepidocrocite, hematite and goethite as the weathering products of primary Fe–bearing phases with the predominance of hematite and goethite over magnetite at later stages of weathering. Transformation of magnetite to goethite is supported by highly alkaline environment and aerobic conditions in MSWI ashes [66]. The alteration of Fe–bearing phases was also observed during the incineration process. Li et al. [67] described the transformation of magnetite to maghemite (275–375 °C) and hematite (590–650 °C) during the incineration. Although jarosite is a mineral frequently found in acidic environments rich in sulphates [68], it was detected in our MSWI ash sample (Fig. 2, Fig. 3). According to Ref. [69], the formation of jarosite and schwertmannite could be induced by bacterial oxidation of Fe^2+^. The occurrence of bone fragments in the incinerated waste resulted in high apatite content (Fig. 2, Fig. 3). Calcite and albite were found in the weathered MSWI ash. The high proportion of calcite in MSWI ashes is due to the carbonation of fresh MSWI ashes and the intense precipitation in the early and advanced stages of weathering, respectively [6,9,12,18]. Grains of calcite and albite originating from building materials, mostly masonry, were identified by Raman spectroscopy. Pieces of building materials might come either from new deposits of building waste that were found during the area inspection or might be a part of incinerated waste residues. The occurrence of sylvite in the sample could be explained by its dissolution and subsequent precipitation as a result of alternating wet and dry conditions [70]. Raman spectroscopy found the efflorescent mineral mascagnite (Fig. 3), which is commonly present in other types of waste, e.g. coal–waste heaps [71]. An interesting aspect was the relatively frequent occurrence of secondary Cu minerals like malachite, brochantite and aurichalcite (Fig. 2, Fig. 3). Oxidising agents, e.g. dissolved alkyl, free chlorine and monochloramine, are able to corrode metallic copper. Newly formed Cu_2_O and CuOH are further oxidised to form Cu(II) solid species [72].

**Table 1:** Quantitative abundance of mineral phases in weathered MSWI ash-rich sample S9a before leaching (original sample) and after leaching in 1 mM citric acid (CA) and oxalic acid (OA) solutions determined by XRD analysis and mineral phases identified by Raman spectroscopy in the original sample. Major minerals are highlighted in bold.

Mineral phase	Chemical formula	XRD analysis			
		Original sample	CA sample	OA sample	Raman spectroscopy
albite	NaAlSi_3_O_8_	n.d.^a^	n.d.	n.d.	X^b^
**apatite**	Ca_10_(PO_4_)_6_(OH)_2_	>10	<2^c^	<2	X
aurichalcite	(Zn,Cu)_5_(CO_3_)_2_(OH)_6_	n.d.	n.d.	n.d.	X
brochantite	Cu_4_(SO_4_) (OH)_6_	n.d.	n.d.	n.d.	X
**calcite**	CaCO_3_	2–10	2–10	2–10	X
caracolite	Na_3_Pb_2_(SO_4_)_3_Cl	<2	n.d.	n.d.	n.d.
cornetite	Cu_3_PO_4_(OH)_3_	<2	<2	n.d.	n.d.
covellite	CuS	<2	<2	n.d.	n.d.
goethite	FeO(OH)	n.d.	n.d.	n.d.	X
gordaite	NaZn_4_(SO_4_) (OH)_6_Cl·6H_2_O	<2	<2		n.d.
**hematite**	α-Fe_2_O_3_	<2	n.d.	>10	X
jarosite	KFe^3+^_3_(SO_4_)_2_(OH)_6_	n.d.	n.d.	n.d.	X
lazurite	Na_3_Ca(Al_3_Si_3_O_12_)S_2_	n.d.	n.d.	n.d.	X
**magnetite**	Fe^2+^Fe^3+^_2_O_4_	n.d.	>10	n.d.	X
**maghemite**	γ-Fe_2_O_3_	>10	n.d.	n.d.	n.d.
malachite	Cu_2_(CO_3_) (OH)_2_	n.d.	n.d.	n.d.	X
mascagnite	(NH_4_)_2_SO_4_	n.d.	n.d.	n.d.	X
melilite	(Ca,Na)_2_(Al,Mg,Fe^2+^)[(Al,Si)SiO_7_]	<2	n.d.	n.d.	n.d.
**mullite**	Al_2_0_3_·2SiO_2_ to 2Al_2_O_3_·SiO_2_	2–10	<2	>10	X
nukundamite	Cu_3·4_Fe_0·6_S_4_	<2	n.d.	n.d.	n.d.
phlogopite	KMg_3_(Si_3_Al)O_10_(F,OH)_2_	n.d.	n.d.	n.d.	X
retgersite	NiSO_4_·6(H_2_O)	n.d.	n.d.	<2	n.d.
rutile	TiO_2_	n.d.	n.d.	n.d.	X
sylvite	KCl	<2	<2	<2	n.d.
**quartz**	SiO_2_	>10	2–10	2–10	X
vaterite	CaCO_3_	n.d.	n.d.	>10	n.d.
wurtzite	(Fe,Zn)S	<2	n.d.	<2	n.d.
**Amorphous phases** (silicate glasses, Fe (hydr)oxides)		>10	>10	>10	X

a Not determined.

b Confirmed by Raman spectroscopy.

c Presence of mineral phases with quantitative abundance <2 wt%, which are not confirmed by other method is questionable.

### 3.3 Leachate chemical composition

The water–MSWI ash suspensions were slightly alkaline, with pH ranging from 7.72 to 8.37 (Table 2), which is close to calcite equilibrium [26]. The addition of citric and oxalic acids had no significant effect on the final pH values of the leachates (single factor ANOVA, F_4.25_ = 1.94, *p* = NS), indicating a high buffering capacity of MSWI ash samples. All leachates were close to saturation with calcite (SI = 0.10–1.07), which documented its primary role in regulating acid–base conditions, i.e. pH of leachates formed during the interaction of weak organic acid solutions with MSWI ashes. On the other hand, fresh MSWI ashes are commonly alkaline with pH values between 10 and 12 [9,25] but due their progressive weathering and extensive calcite precipitation, pH of MSWI ashes decreases to 7–9 [20]. As discussed above, calcite was abundant in the MSWI ash sample (Table 1).

**Table 2:** Values of pH in soil-water suspensions and leachates, dissolved concentrations of Fe and trace metals in the leachates (μg/L) after extraction with low molecular weight organic acids and dissolved fractions in % (shown in parentheses).

	Treatment	Samples					
		S7	S8	S9a	S9b	S10a	S10b
pH(H_2_O)		7.72	8.15	8.19	7.97	8.37	8.18
Final pH	1.0 mM CA^a^	8.00	8.13	8.21	8.00	8.25	8.08
	0.1 mM CA	7.82	8.00	8.17	7.94	8.23	8.22
	1.0 mM OA^a^	8.14	8.30	8.14	7.92	8.18	7.96
	0.1 mM OA	8.10	8.23	8.01	7.99	8.29	8.19
Ba	1.0 mM CA	179 (0.12)	123 (0.07)	91.6 (0.03)	49.1 (0.02)	98.4 (0.04)	66.9 (0.03)
	0.1 mM CA	96.7 (0.06)	83.9 (0.05)	76.0 (0.03)	51.8 (0.02)	78.0 (0.03)	70.1 (0.03)
	1.0 mM OA	125 (0.08)	96.9 (0.06)	83.2 (0.03)	52.9 (0.02)	77.1 (0.03)	59.8 (0.02)
	0.1 mM OA	110 (0.07)	82.8 (0.05)	81.9 (0.03)	66.2 (0.03)	89.1 (0.03)	86.7 (0.04)
Cd	1.0 mM CA^b^	**16.6** (0.55)^c^	**5.30** (0.53)	**9.00** (0.47)	**6.90** (0.58)	**7.00** (0.50)	**8.50** (0.47)
Cr	1.0 mM CA	4.50 (0.01)	8.10 (0.03)	<2.0	<2.0	<2.0	<2.0
	1.0 mM OA	2.20 (0.01)	<2.0	<2.0	<2.0	<2.0	<2.0
	0.1 mM OA	3.80 (0.01)	<2.0	<2.0	<2.0	2.50 (0.01)	<2.0
Cu	1.0 mM CA	**235** (0.15)	**333** (0.36)	**688** (0.39)	**965** (0.39)	**446** (0.28)	**524** (0.33)
	0.1 mM CA	152 (0.09)	25.0 (0.03)	14.1 (0.01)	19.6 (0.01)	13.2 (0.01)	12.6 (0.01)
	1.0 mM OA	150 (0.09)	17.0 (0.02)	7.70 (0.00)	11.0 (0.00)	9.20 (0.01)	12.3 (0.01)
	0.1 mM OA	168 (0.10)	20.5 (0.02)	8.10 (0.00)	8.10 (0.00)	7.30 (0.00)	10.1 (0.01)
Fe	1.0 mM CA	622 (0.01)	416 (0.01)	292 (0.00)	260 (0.00)	308 (0.00)	289 (0.00)
	0.1 mM CA	64.3 (0.00)	21.2 (0.00)	<5.0	<5.0	10.7 (0.00)	<5.0
	1.0 mM OA	124 (0.00)	22.3 (0.00)	<5.0	5.30 (0.00)	5.30 (0.00)	<5.0
	0.1 mM OA	212 (0.00)	14.7 (0.00)	<5.0	<5.0	<5.0	<5.0
Mn	1.0 mM CA	173 (0.09)	74.2 (0.07)	39.3 (0.02)	96.2 (0.05)	54.8 (0.03)	82.6 (0.04)
	0.1 mM CA	3.60 (0.00)	1.40 (0.00)	5.50 (0.00)	20.4 (0.01)	2.60 (0.00)	11.1 (0.00)
	1.0 mM OA	19.7 (0.01)	6.40 (0.01)	17.9 (0.01)	49.1 (0.02)	5.80 (0.00)	33.8 (0.02)
	0.1 mM OA	12.9 (0.01)	1.50 (0.00)	5.20 (0.00)	16.3 (0.01)	2.10 (0.00)	12.0 (0.01)
Ni	1.0 mM CA	14.4 (0.21)	**47.7** (0.35)	30.1 (0.17)	25.3 (0.18)	23.6 (0.14)	**57.9** (0.19)
	0.1 mM CA	7.00 (0.10)	<5.0	<5.0	<5.0	<5.0	<5.0
	1.0 mM OA	5.10 (0.07)	<5.0	<5.0	<5.0	<5.0	<5.0
Pb	1.0 mM CA	34.0 (0.02)	<0.5	<0.5	<0.5	<0.5	<0.5
	1.0 mM OA	15.0 (0.01)	<0.5	<0.5	<0.5	<0.5	<0.5
	0.1 mM OA	26.0 (0.02)	<0.5	<0.5	<0.5	<0.5	<0.5
Zn	1.0 mM CA	**1410** (0.17)	**741** (0.14)	**973** (0.09)	**782** (0.09)	**607** (0.06)	**600** (0.07)
	0.1 mM CA	160 (0.02)	25.2 (0.00)	31.8 (0.00)	68.3 (0.01)	22.6 (0.00)	27.0 (0.00)
	1.0 mM OA	261 (0.03)	40.7 (0.01)	139 (0.01)	184 (0.02)	28.3 (0.00)	76.6 (0.01)
	0.1 mM OA	279 ((0.03)	13.1 (0.00)	33.7 (0.00)	55.1 (0.01)	13.7 (0.00)	36.2 (0.00)

a CA = citric acid solution, OA = oxalic acid solution.

b Missing LMWOA treatment for the chemical parameter means that the metal concentrations in the leachates of all six MSWI samples were below the detection limit.

c Concentrations of metals (Cd, Cu, Ni and Zn) in the leachates marked in bold type exceeded the criteria for inert waste (4.00, 200, 40.0 and 400 μg/L for Cd, Cu, Ni and Zn, respectively) according to the legislation of the European Union [74].

The leached metal (loid) concentrations and their percentage fractions are listed in Table 2. The extraction results for other elements (Al, Ca, K, Mg, Na) and anions (Cl^−^, SO_4_^2−^, HCO_3_^−^) are shown in Table S5. The concentrations of As and Sb in the leachates from all samples and treatments were below the detection limit of the analytical method (<5.0 μg/L), so they are not discussed further. Aluminium, Cu, Fe, Mn and Zn showed statistically significant differences in dissolved concentrations among the four treatments, while citric acid at a concentration of 1 mM leached the highest amount of metals. Although the ANOVA test for Cd, Cr, Ni and Pb could not be reliably used due to many concentration data below the detection limits, the highest leached concentrations were observed for treatments with 1 mM citric acid solution (Table 2). These findings were in agreement with the results of previous studies that compared the effect of several LMWOAs on the mobility of trace metal (loid)s in solid environmental matrices [[29], [30], [31],^41^]. The final pH values of the leachates were approximately the same for all four LMWOA treatments, and therefore, pH was not the main factor controlling metal release (Table 2). Considering the dissociation constants of these organic acids (1.27 and 4.28 for oxalic acid; 3.13, 4.76 and 6.40 for citric acid) and the acid–base conditions in the leachates (pH ≈ 8), they occurred as organic anions capable of forming complexes with metal cations. The increased effect of citric acid on the metal leaching could be explained by the fact that it contains three carboxyl groups, while oxalic acid has two carboxyl groups. Citric acid can therefore form tridentate ligands (six-membered ring structures) with metals; larger complex compounds that are usually more stable and more mobile than the smaller bidentate metal-oxalate ligands [38,73]. The stability of organic acid complexes with metals is indicated by their respective stability constants, being mostly higher for citrate complexes than for oxalate complexes [41].

Although the European legislation on criteria for inert waste is based on the leachability of chemical elements in deionised water [74], its application can also be useful for leaching from aqueous solutions of LMWOAs. The concentrations of Cd, Cu and Zn in all samples, Ni in two samples extracted with 1 mM citric acid solution, Cl^−^ in one sample and SO_4_^2−^ in three samples extracted with both organic acids at both concentration levels exceeded the respective threshold values for inert waste (Table 2 and S5) but at lower, more realistic concentrations of organic acids, dissolved concentrations of metals were below the guideline values of inert waste. The fact that these weathered MSWI residues are chemically unreactive even when attacked with organic acids, at least, as observed in the short-term leaching tests performed in this study, was reflected in the leached metal proportions (the ratio of the leached metal concentration to its total concentration), which did not exceed 1% and ranged from tenths of % (only Cd, Cu, Ni and Zn extracted with 1 mM citric acid) to less than thousandths of % (Table 2).

The results of the speciation calculations for trace metals are given in Table 3. The speciation of the major elements (Al, Ca, Fe, K, Mg, Na) is shown in Table S6. The metal speciation in the leachates clearly depended on the concentration and type of LMWOAs as well as on the metal. Visual MINTEQ 3 predicted that the predominant form of Cd was the free Cd^2+^ ion in all citric acid treatments, followed by alternating proportions of sulphate (CdSO_4_^0^ and Cd(SO_4_)_2_^2−^) and carbonate (CdCO_3_^0^ and CdHCO_3_^+^) species, while the proportion of organic Cd-citrate^–^ (8.29–20.4%) was several times higher at a citric acid concentration of 1 mM than at 0.1 mM (1.15–6.21%). However, in leachates with 1 mM oxalic acid solution, Cd-oxalate^0^ prevailed over Cd sulphate and carbonate species. Similar differences, especially in the proportions of citrate and oxalate species, were also observed for other metals, being significantly higher at 1 mM concentration of the respective LMWOAs. The higher proportions of citrate- and oxalate-metal complexes in the leachates of 1 mM LMWOA solutions compared to those in the leachates of 0.1 mM LMWOA solutions (Table 3) could explain the higher leachability of metals from solid samples extracted with 1 mM LMWOA solutions (Table 2). Anionic and neutral organo–metal complexes are not significantly adsorbed in weakly alkaline soils due to the predominant, negatively charged surfaces of organic matter, clay minerals and Al, Fe and Mn (hydr)oxides [75].

**Table 3:** The main dissolved species of trace metals in the leachates from weathered MSWI ashes. Values are expressed in % and represent arithmetic mean ± standard deviation from six samples. Only species with fractions above 1% are shown.

Species	1 mM citric acid	0.1 mM citric acid	Species	1 mM oxalic acid	0.1 mM oxalic acid
Ba^2+^	81.4 ± 7.60	84.2 ± 9.56	Ba^2+^	83.0 ± 8.79	85.1 ± 8.80
BaSO_4_^0^	11.6 ± 9.35	16.2 ± 8.78	BaSO_4_^0^	15.6 ± 8.79	15.7 ± 7.74
BaHCO_3_^+^	2.43 ± 0.83	1.25 ± 0.62	BaHCO_3_^+^	1.52 ± 0.64	1.15 ± 0.64
Ba-citrate^–^	3.76 ± 1.29	<1	Ba-oxalate^0^	1.77 ± 0.72	<1
Cd^2+^	46.6 ± 4.04	61.0 ± 12.3	Cd^2+^	40.4 ± 1.44	58.9 ± 7.90
CdCl^+^	2.89 ± 4.27	3.41 ± 4.54	CdCl^+^	2.72 ± 4.11	3.46 ± 4.75
CdSO_4_^0^	11.8 ± 10.2	19.5 ± 12.4	CdSO_4_^0^	14.5 ± 10.6	19.0 ± 11.7
Cd(SO_4_)_2_^2–^	1.28 ± 1.83	1.76 ± 2.23	Cd(SO_4_)_2_^2–^	1.42 ± 1.86	1.44 ± 1.65
CdHCO_3_^+^	4.60 ± 1.53	2.97 ± 1.53	CdHCO_3_^+^	2.41 ± 0.88	2.59 ± 1.34
CdCO_3_^0^	18.2 ± 5.22	10.6 ± 4.54	CdCO_3_^0^	10.0 ± 4.81	11.6 ± 6.17
Cd-citrate^–^	13.8 ± 4.60	3.51 ± 2.06	Cd-oxalate^0^	30.5 ± 9.74	5.67 ± 2.49
Cu^2+^	<1	1.62 ± 0.91	Cu^2+^	<1	1.84 ± 1.21
CuOH^+^	1.29 ± 0.20	4.00 ± 1.25	CuOH^+^	<1	5.25 ± 1.79
CuCO_3_^0^	42.5 ± 5.29	58.8 ± 9.08	CuCO_3_^0^	12.1 ± 4.37	70.0 ± 9.95
Cu(CO_3_)_2_^2–^	5.13 ± 1.72	3.53 ± 3.80	Cu(CO_3_)_2_^2–^	<1	3.84 ± 3.06
Cu-citrate^–^	50.0 ± 6.56	30.5 ± 11.7	Cu-(oxalate)_2_^2–^	76.1 ± 5.17	9.52 ± 4.52
			Cu-oxalate^0^	9.98 ± 0.83	10.6 ± 4.68
Ni^2+^	6.69 ± 1.71	24.9 ± 6.41	Ni^2+^	4.93 ± 1.80	28.6 ± 4.44
NiSO_4_^0^	1.74 ± 1.95	8.73 ± 7.87	NiSO_4_^0^	1.83 ± 1.72	9.12 ± 6.94
NiCO_3_^0^	3.95 ± 0.92	7.05 ± 4.94	NiCO_3_^0^	1.74 ± 0.64	8.66 ± 4.88
NiHCO_3_^+^	2.55 ± 0.90	4.70 ± 2.54	NiHCO_3_^+^	1.11 ± 0.49	4.84 ± 2.58
Ni-citrate^–^	84.4 ± 4.20	55.7 ± 16.2	Ni-(oxalate)_2_^2–^	28.2 ± 3.45	2.51 ± 0.88
			Ni-oxalate^0^	62.4 ± 1.81	47.4 ± 10.6
Mn^2+^	38.9 ± 2.67	58.4 ± 7.96	Mn^2+^	37.9 ± 4.87	56.7 ± 4.88
MnSO_4_^0^	8.11 ± 7.86	15.2 ± 9.95	MnSO_4_^0^	11.1 ± 8.39	14.9 ± 9.09
MnCO_3_^0^	31.8 ± 6.88	21.6 ± 9.20	MnCO_3_^0^	19.3 ± 7.54	23.1 ± 11.1
MnHCO_3_^+^	2.39 ± 0.68	1.76 ± 0.81	MnHCO_3_^+^	1.40 ± 0.45	1.53 ± 0.68
Mn-citrate^–^	18.7 ± 4.68	5.42 ± 2.91	Mn-oxalate^0^	31.8 ± 7.58	6.11 ± 2.29
Pb^2+^	1.53 ± 0.54	3.69 ± 1.79	Pb^2+^	2.06 ± 1.03	3.51 ± 2.29
PbOH^+^	3.41 ± 0.92	7.37 ± 2.43	PbOH^+^	4.35 ± 0.82	8.01 ± 2.89
PbSO_4_^0^	1.04 ± 1.34	3.29 ± 3.84	PbSO_4_^0^	2.08 ± 2.22	3.40 ± 3.88
Pb(CO_3_)_2_^2–^	8.96 ± 1.99	4.07 ± 3.55	Pb(CO_3_)_2_^2–^	4.01 ± 1.47	3.90 ± 2.84
PbCO_3_^0^	79.2 ± 2.12	76.8 ± 5.73	PbCO_3_^0^	60.7 ± 7.18	73.1 ± 8.83
PbHCO_3_^+^	3.57 ± 0.84	3.95 ± 1.55	PbHCO_3_^+^	2.79 ± 0.86	3.05 ± 0.67
Pb-citrate^–^	2.09 ± 0.54	<1	Pb-oxalate^0^	23.5 ± 5.36	5.11 ± 2.34
Zn^2+^	16.9 ± 3.11	38.1 ± 5.94	Zn^2+^	10.2 ± 3.20	33.8 ± 4.43
ZnOH^+^	1.50 ± 0.18	3.21 ± 0.72	ZnOH^+^	<1	3.39 ± 0.71
Zn(OH)_2_^0^	2.28 ± 0.86	4.71 ± 2.47	Zn(OH)_2_^0^	1.40 ± 0.70	5.69 ± 2.77
ZnSO_4_^0^	4.61 ± 4.92	12.9 ± 10.3	ZnSO_4_^0^	4.04 ± 3.66	11.6 ± 8.63
ZnCO_3_^0^	15.6 ± 3.37	16.1 ± 7.71	ZnCO_3_^0^	5.62 ± 2.01	15.7 ± 8.03
ZnHCO_3_^+^	1.65 ± 0.54	1.84 ± 0.92	Zn-(oxalate)_2_^2–^	10.6 ± 1.89	<1
Zn-citrate^–^	56.9 ± 6.87	24.4 ± 10.9	Zn-oxalate^0^	67.1 ± 4.17	29.2 ± 7.87

The minerals detected in the sample S9a after LMWOA leaching are listed in Table 1. Changes in proportions of minerals were likely due to the high sample heterogeneity and uneven distribution of mineral phases in MSWI ash. The XRD patterns did not change significantly after the leaching of the MSWI ash with citric and oxalic acids (Figure S4). Banerjee et al. [76] observed no destruction of mineral phases after the coal ash treatment with carboxylic acids, even though much higher LMWOA concentrations were used when compared to those used in this study. However, prolonged exposure to LMWOAs can cause accelerated weathering of soil and ash minerals, followed by the release of trace metal (loid)s into the pore solution [77,78].

Several solid phases controlling the solubility of major and trace metal (loid)s in weathered MSWI ashes were predicted (Table S7). Consistent with the mineralogical observation of abundant Fe (hydr)oxides, ferrihydrite showed highly positive SI values in all leachates and leachates using 1 mM citric acid solution were supersaturated with respect to K-jarosite, which was found in the ash sample subjected to mineralogical inspection (sample S9a). Moreover, all leachates were supersaturated with respect to goethite, hematite and maghemite. Calcite, another commonly identified mineral (Table 1), was predicted to be in equilibrium or to precipitate, while calcium oxalate trihydrate (CaC_2_O_4_·3H_2_O) was saturated/slightly supersaturated in oxalic acid leachates. The mineral partly controlling the solubility of Cu appears to be brochantite because the 1 mM citric acid leachates were saturated/slightly supersaturated with respect to this mineral and brochantite was identified in the MSWI ash sample (Fig. 2, Fig. 3). According to equilibrium calculations, malachite as another mineral limiting the Cu mobility, precipitated or was close to equilibrium in the case of leaching with citric acid, which agreed with its presence in the ash sample S9a and findings of Dijkstra et al. [21]. Smithsonite (ZnCO_3_) and otavite (CdCO_3_) were predicted to potentially control the extractability of Zn and Cd with citric acid solution, respectively, but none of these minerals were found. According to equilibrium calculations, leachates from all treatments were close to equilibrium or supersaturated with respect to barite (Table S7), which likely explains the low solubility of Ba [79,80]. Although the chemistry of the detected mineral phases was not studied, it could be assumed that Cd, Zn and Ni were released preferentially from calcite, which effectively incorporates these metals through several mechanisms [63,81]. Leaching of these metals from crystalline and amorphous Fe (hydr)oxides could not be ruled out [63,82], however, very low Fe concentrations in the leachates (Table 2) and significant supersaturation of leachates with Fe (hydr)oxides (Table S7) indicated their negligible solubility under the conditions of the leaching experiment.

### 3.4 Human health and ecological risk assessment, and environmental implications

Human health risks from exposure to soil contaminants were assessed for construction workers in the study area over 2 years, considering three main exposure routes: (i) soil ingestion, (ii) inhalation of soil particles, and (iii) dermal contact (Table 4). The missing values in Table 4 indicate that there are no RfD or SF values for given exposure routes. The total hazard index (HI_total_) calculated as the sum of the HI values of individual metal (loid)s across all three exposure routes was lower than 1. Therefore, the area is not a risk factor to the human health of construction workers likely due to short exposure duration of two years. Soil ingestion contributed the most to the total non-carcinogenic health risk (92.0%), followed by inhalation of soil particles (4.2%) and dermal contact (3.8%), copying the findings of many recent studies [[83], [84], [85]]. Among the trace metal (loid)s, Pb, Sb and As from soil ingestion showed the greatest contribution to the non-carcinogenic health risk for workers (45.9%, 23.2% and 7.6% of the HI_total_ value, respectively), while the other metal (loid)s contributed considerably less. However, it is important to emphasise that the HI_total_ value was based on a conservative assumption of 100% metal (loid) bioavailability, which is actually not achievable. Bioavailability of metal (loid)s in soil is usually lower than 100%, e.g. it is typically around 60%, 20% and 30–40% for As, Ni and Pb, respectively, as shown by in vivo mammalian tests [[86], [87], [88], [89]], therefore, HI_total_ value would be lower than the estimated value of 0.67. Carcinogenic health risk was not confirmed as the total risk value (CR_total_) was much lower than the default value of 10^−4^, below which no significant risk of cancer is assumed (Table 4). It should be noted that MSW incinerators and their solid products (fly and bottom ashes) can be a source of other harmful compounds, especially dioxins (collective name for polychlorinated dibenzo-*p*-dioxins and dibenzofurans) [90]; an aspect not studied here. However, strict EU legislation has led to a significant reduction in dioxin emissions, showing that the entire waste sector, including also incineration of wastes other than municipal, is currently responsible for about one fourth of the total anthropogenic dioxin emissions [91,92]. Therefore, their occurrence in the sampled MSWI ash and soil is likely, which would lead to an increase in HI_total_ and CR_total_ values. However, most studies have not demonstrated non-carcinogenic and carcinogenic hazards from human exposure to dioxins in soil or MSWI incinerator emissions [[93], [94], [95], [96]], and there is still ongoing debate about their effects on human health outcomes [[97], [98], [99]].

**Table 4:** Estimation of non-carcinogenic and carcinogenic human health risks for trace metal (loid)s in soils. The values were calculated from upper confidence limit values of metal (loid) concentrations.

	As	Ba	Cd	Cr	Cu	Mn	Ni	Pb	Sb	Zn	HI_route_^b^	HI_total_^c^
*non-carcinogenic risk*												
HQ_ingestion_	5.05 × 10^−2^	7.47 × 10^−3^	2.05 × 10^−2^	1.47 × 10^−4^	3.46 × 10^−2^	1.15 × 10^−2^	1.02 × 10^−2^	3.05 × 10^−1^	1.55 × 10^−1^	1.71 × 10^−2^	0.613	
HQ_inhalation_	5.94 × 10^−4^	1.76 × 10^−3^	1.21 × 10^−3^	2.59 × 10^−5^	NA^a^	1.90 × 10^−2^	4.73 × 10^−3^	NA	1.22 × 10^−4^	NA	0.027	
HQ_dermal_	6.75 × 10^−3^	3.16 × 10^−5^	3.47 × 10^−3^	4.77 × 10^−5^	2.57 × 10^−4^	8.12 × 10^−4^	1.08 × 10^−3^	8.62 × 10^−3^	4.39 × 10^−3^	3.61 × 10^−4^	0.026	**0.666**
	As	Ba	Cd	Cr	Cu	Mn	Ni	Pb	Sb	Zn	CR_route_^b^	CR_total_^c^
*carcinogenic risk*												
CR_ingestion_	6.49 × 10^−7^	NA	NA	NA	NA	NA	NA	2.67 × 10^−7^	NA	NA	9.16 × 10^−7^	
CR_inhalation_	1.10 × 10^−9^	NA	6.21 × 10^−10^	4.43 × 10^−8^	NA	NA	4.54 × 10^−10^	2.22 × 10^−10^	NA	NA	4.67 × 10^−8^	
CR_dermal_	8.68 × 10^−8^	NA	NA	NA	NA	NA	NA	5.65 × 10^−9^	NA	NA	9.25 × 10^−8^	**1.06**×**10**^**−**^**^6^**

a Not available.

b HI_route_ or CR_route_ = the sum of the HQ or CR values of all metal (loid)s for one exposure route.

c HI_total_ or CR_total_ = the sum of all three HI_route_ or CR_route_ values.

**Fig. 4:**
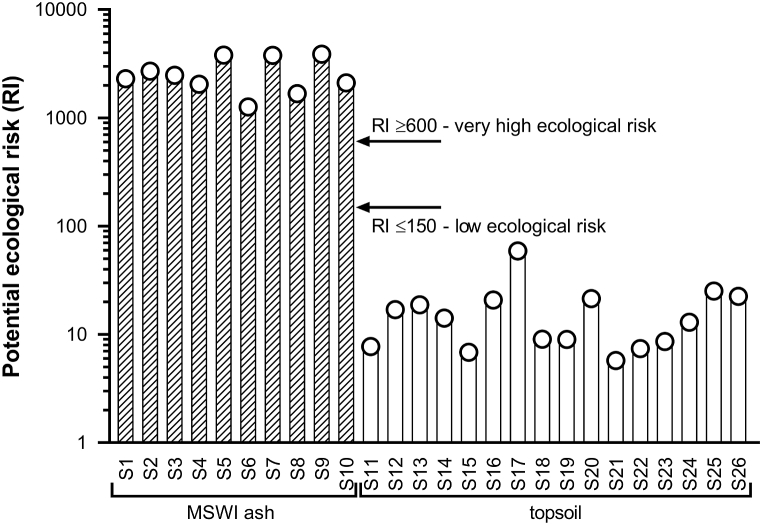
Assessed ecological risk of individual sampled points in the study area with freely dumped MSWI ash residues.

Ecological risks posed by trace metal (loid)s were very high (RI ≥ 600) (Fig. 4), but limited only to the area covered by MSWI ashes, corresponding to samples S1–S10 (Figure S1). The remaining area had a low ecological risk with RI values considerably lower than 150. The prominent metal (loid)s contributing to very high ecological risk were Cd (mean *E*_r_ value of 1542), followed by Sb, Pb and Cu with the mean *E*_r_ values of 660, 435 and 404, respectively.

As shown by the results described in section 3.1, the study area was contaminated with As, Ba, Cd, Cr, Cu, Mn, Ni, Pb, Sb and Zn. According to the values of EF, the soils were categorised as significantly up to extremely enriched with Cd, Cu, Pb, Sb and Zn (Fig. 1c), and most of the trace metal (loid)s exhibited total concentrations exceeding the respective ID or even IT values. It could be concluded that the area should be remediated according to the Slovak legislation on environmental burdens. Remediation of the area has already been performed by mechanical excavation of contaminated soils, but the covering of soils with MSWI ashes is still observable in many places. However, when other results were taken into account, i.e. mineralogy of MSWI residues, leaching tests with diluted LMWOA solutions and groundwater chemistry, along with the size of area and its industrial land-use, actual environmental and human health risks seem to be low. The buried MSWI ashes represent a material in a high stage of weathering with the presence of stable mineral phases and buffering capacity, which was also confirmed by the low leached concentrations of harmful chemical elements and the increase of leachate pH to the alkaline range, naturally decreasing the mobility of trace metals. The sampling of groundwater from five wells located in the study area showed that its chemical composition was not affected by the deposited MSWI ashes (Table 5, Table 6). Concentrations of dissolved trace metal (loid)s were low, frequently below the detection limits of the analytical method used. The groundwater was of Ca–Na-(Mg)/HCO_3_–Cl-(SO_4_) type with a mean pH of 7.49 and total dissolved solids of 785 mg/L (Table 6). The quality and hydrogeochemistry of groundwater within the area did not differ from those outside the landfill, which are regularly analysed by the Slovak Hydrometeorological Institute as part of the groundwater monitoring in Slovakia. Trace metal (loid) concentrations were almost identical (Table 5), only chlorides in groundwater from the area were approximately two to five times higher than those in groundwater from monitoring wells, except for well 273,190 (Table 6). Higher Cl^−^ concentrations in groundwater from the sampled well and monitoring well 273,190 (at the airport) may be related to the use of technical salts during winter maintenance of the road, passing close to the landfill, and airport runways, respectively. It should be noted that the concentrations of all major ions and trace metal (loid)s did not exceed their respective limit values for drinking water, except for Fe and Mn. However, these two metals do not pose a significant health risk. Increased concentrations of Fe and Mn in groundwater are related to redox conditions and insufficient saturation of groundwater with oxygen in several areas of Bratislava city [102].

**Table 5:** Range of trace metal (loid) concentrations in the groundwater of the study area from five wells sampled in April–September 2020 and comparison to their concentrations in the groundwater outside the area from five monitoring wells of the Slovak Hydrometeorological Institute (SHMI).

	μg/L							
	As	Cd	Cr	Cu	Ni	Pb	Sb	Zn
the area	<5.0	<0.1	<2.0–2.5	<2.0	<2.0–4.2	<0.5	<5.0	<2.0–15.4
outside the area^a^	<0.5–1.3	<0.1	<2.0	<2.0–3.0	<2.0–2.0	<0.5–10.9	<0.5	<2.0–19.0
drinking water limit^b^	10	5.0	50	2000	20	10	5.0	na^c^

a Publicly available data on the website of the Slovak Hydrometeorological Institute (https://www.shmu.sk/en/?page=2451); the concentration range shown in the table is a data summary from five monitoring wells located in Bratislava District II for the years 2020–2021.

b Council Directive 98/83/EC of November 3, 1998 on the quality of water intended for human consumption [100].

c Not available.

**Table 6:** Major ion chemistry of groundwater sampled from the well GW-1 located in the study area and monitoring wells outside the area managed by the Slovak Hydrometeorological Institute (SHMI). Results are shown as arithmetic mean ± standard deviation and all concentration data are in mg/L.

	drinking water limit^b^	GW-1	monitoring wells outside the area^a^				
			720,190	720,090	601,692	272,690	273,190
pH	6.5–9.5	7.49 ± 0.41	7.61 ± 0.08	7.32 ± 0.12	7.45 ± 0.12	7.27 ± 0.29	7.17 ± 0.03
Eh (mV)	–	165 ± 30.9	197 ± 25.8	376 ± 74.7	280 ± 81.4	335 ± 120	336 ± 40.6
Total dissolved solids	–	785 ± 91.6	416 ± 35.2	617 ± 51.8	478 ± 46.1	691 ± 74.5	918 ± 86.2
Na	200	78.3 ± 8.13	35.5 ± 5.24	34.1 ± 13.3	13.3 ± 4.48	25.2 ± 0.80	69.2 ± 3.02
K	–	6.99 ± 0.58	2.50 ± 0.29	6.20 ± 1.83	3.73 ± 1.48	4.65 ± 0.10	9.23 ± 0.40
Ca	>30	106 ± 12.3	60.5 ± 1.28	94.4 ± 20.9	80.5 ± 13.3	112 ± 2.38	126 ± 5.45
Mg	125	24.5 ± 2.77	12.5 ± 0.67	22.0 ± 4.68	19.0 ± 3.62	27.8 ± 1.35	34.9 ± 2.37
Mn	0.05	**0.85** ± 0.28	**0.20** ± 0.08	0.01 ± 0.00	0.02 ± 0.02	0.00 ± 0.00	**0.23** ± 0.03
Fe	0.20	**0.78** ± 0.60	**0.81** ± 0.39	0.04 ± 0.06	0.08 ± 0.14	0.01 ± 0.01	0.01 ± 0.00
HCO_3_^−^	–	372 ± 54.8	221 ± 7.27	323 ± 54.3	276 ± 40.3	416 ± 11.4	461 ± 8.09
SO_4_^2−^	250	73.6 ± 8.21	34.1 ± 1.65	47.9 ± 13.1	50.2 ± 10.2	62.6 ± 3.19	85.6 ± 3.71
Cl^−^	250	114 ± 10.2	48.6 ± 11.5	66.8 ± 31.1	21.6 ± 2.94	37.8 ± 1.74	117 ± 6.45
NO_3_^−^	50	8.83 ± 2.30	<1.00	23.0 ± 11.6	13.2 ± 5.69	5.13 ± 1.31	15.3 ± 4.05
Hydrogeochemistry^c^		Ca–Na-(Mg)/HCO_3_–Cl-(SO_4_)	Ca–Na-(Mg)/HCO_3_–Cl	Ca–Mg-(Na)/HCO_3_–Cl-(SO_4_)	Ca–Mg/HCO_3_-(SO_4_)	Ca–Mg-(Na)/HCO_3_-(SO_4_)-(Cl)	Ca–Na–Mg/HCO_3_–Cl-(SO_4_)

a Publicly available data on the website of the Slovak Hydrometeorological Institute (https://www.shmu.sk/en/?page=2451); the results shown in the table are a data summary from five monitoring wells located in Bratislava District II for the years 2020–2021.

b Council Directive 98/83/EC of November 3, 1998 on the quality of water intended for human consumption [100] and Decree No. 247/2017 [101].

c Underlined ions are above 50 *c*_i_ × *z*_i_%; ions in parentheses represent the 10–20 *c*_i_ × *z*_i_% interval.

### 3.5 Comparison to other MSWI areas

To put the results of this study in a wider international context, they were compared to previous findings from other areas with MSW incinerators or MSWI ash landfills. Studies of the impact of MSW incinerators on the concentrations of metal (loid)s in soils from several countries of the world indicate a wide range of their concentrations (Table S8), which applies not only to the parameters of the incinerator (e.g. composition and volume of incinerated MSW, operating time of incineration technology, emission control and treatment) but also to local geology, climate and other anthropogenic activity, either historical or recent [58,103]. On the other hand, the assumption that the activity of the MSW incinerator has an effect on the concentration of metal (loid)s in soils is not always valid, which is in line with the results of this study, i.e. high concentrations of metal (loid)s are related to MSWI ash deposited in soil and their concentrations in surrounding soil are similar to the respective backgrounds. For example, Rovira et al. [94,95], Rimmer et al. [104], Richardson [105] and Vilavert et al. [106] are of the opinion that MSW incinerators have a low impact on metal (loid) concentrations in soil mainly due to the implementation of advanced air pollution control systems and the separation of waste enriched with metal (loid)s. However, it appears that metal (loid) contamination of soils in the vicinity of MSW incinerators is masked by a much greater effect of other metal (loid) sources, such as traffic emissions, industrial production, domestic heating and historical pollution resulting from previous land-use as shown by Ref. [104]. An interesting finding of the study by Wei et al. [58], reviewing metal (loid) concentrations in soils near MSW incinerators in China, is that although some metals (Cd, Hg) show enrichment, it is comparable to their enrichment in cultivated soils. This shows the ambiguity of the influence of MSW incinerators on the accumulation of metal (loid)s in soils and the parallel action of multiple sources.

To the best of our knowledge, few studies have addressed metal (loid) concentrations in soils containing or in contact with waste incinerator ashes [[107], [108], [109], [110], [111], [112]]. Similar to the results of this study, these technogenic materials are enriched in metal (loid)s (Table S8 – note the striking coincidence of metal (loid) concentrations in MSWI ashes deposited/buried in soils from different countries), while metal (loid) concentrations in the surrounding or overlying soil are usually lower and dependent on the distance from the ash deposits and the ash content of the soil [107,112]. Several authors characterised the mineralogical composition of old MSWI ashes and showed that quartz, calcite, magnetite, hematite and possibly albite were the dominant minerals [51,63,[109], [110], [111]], which is also a typical feature of the old MSWI ashes of this study. Limited leaching of metal (loid)s is characteristic of incineration ashes exposed to atmospheric conditions for a long time. Although metal leaching was determined by different methods with different extractants in the studies, they all highlighted relatively low mobile concentrations of metals [108,111,112], which is consistent with the leaching results presented here. Interestingly, Rigo et al. [111] determined the exchangeable fraction of metals in 35-year-old deposited MSWI ashes to be around 5% for Cd and Cu and 0.1–3% for Al, Cr, Fe, Mn, Ni, Pb and Zn using a 0.5 M citric acid solution. These leached fractions are obviously higher than those in this study (Table 2) due to the incomparably higher molar concentration of citric acid (500- to 5000-times higher) used in that study.

## 4 Conclusions

Using the example from the Danube River basin, we pointed at the potential source of a wide range of trace metal (loid)s harmful for the environment, represented here by MSWI ash deposited in the urban soil without control more than 40 years ago. Total concentrations of metal (loid)s in weathered MSWI ash residues were fairly high but serious metal (loid) contamination was not confirmed in the surrounding soils. A very high ecological risk (RI > 600) was found at ten sampling sites, directly linked to the occurrence of MSWI ash. The human health risk assessment showed that Pb was the main soil contaminant with an HQ value of 0.31 for soil ingestion, yet the HI_total_ and CR_total_ values were lower than 1.0 and 10^−6^, respectively, indicating no non-carcinogenic and carcinogenic risks to humans. The negative impact on groundwater chemistry was not demonstrated. Limited trace metal (loid) mobilisation from the MSWI ash residues was determined in the simulated rhizosphere solutions. This observation can be explained by (i) the high buffering capacity maintaining near-neutral pH, (ii) the presence of stable minerals, and (iii) the fact that metal (loid) labile fractions have already been leached out. The mineral transformations were documented by the presence of secondary Cu minerals (malachite and brochantite). This study highlighted that the geochemical research combining multiple methods was an effective approach to evaluate the impact of MSWI ash residues on the environment and humans. Despite the relatively low mobilisation of contaminants, any point sources near a strategic river or important groundwater sources need to be thoroughly investigated. To conclude, inappropriate management of contaminated areas including local environmental burdens (point contamination sources) without an interdisciplinary context of individual environmental compartments and trace metal (loid) geochemical behaviour, may result in a synergistic effect of contaminant migration from several sources into groundwater in the long term.

## Ethics approval and consent to participate

Not applicable.

## Author contribution statement

Tomáš Faragó, Dr: Conceived and designed the experiments; Performed the experiments; Wrote the paper.

Veronika Špirová, Dr: Performed the experiments.

Petra Blažeková: Conceived and designed the experiments; Performed the experiments.

Bronislava Lalinská-Voleková, Dr: Performed the experiments; Analysed and interpreted the data; Wrote the paper.

Juraj Macek, Dr.; Ľubomír Jurkovič, Assoc. Prof: Contributed reagents, materials, analysis tools or data.

Martina Vítková, Assoc. Prof; Edgar Hiller: Analysed and interpreted the data; Wrote the paper.

## Funding statement

Veronika Špirová was supported by Operation Program of Integrated Infrastructure [ITMS2014+: 313021BUZ3].

Prof. Edgar Hiller was supported by 10.13039/501100005357Agentúra na Podporu Výskumu a Vývoja [APVV-21-0212].

## Data availability statement

Data included in article/supp. Material/referenced in article.

## Declaration of interest's statement

The authors declare that they have no known competing financial interests or personal relationships that could have appeared to influence the work reported in this paper.
