# Supplementary material for: Environmental and health impacts assessment of long-term naturally-weathered municipal solid waste incineration ashes deposited in soil—old burden in Bratislava city, Slovakia
Source: Heliyon. 2023 Feb 9;9(3):e13605. doi: 10.1016/j.heliyon.2023.e13605 (PMC9976324; doi:10.1016/j.heliyon.2023.e13605)
Supplement: Multimedia component 1 [file mmc1.docx]

**Supplementary Material**

**for article entitled**

**Environmental and health impacts assessment of long-term naturally-weathered municipal solid waste incineration ashes deposited in soil – old burden in Bratislava city, Slovakia**

Tomáš Faragó ^a^, Veronika Špirová ^a^, Petra Blažeková ^a^, Bronislava Lalinská-Voleková ^b^, Juraj Macek ^a, c^, Ľubomír Jurkovič ^a^, Martina Vítková ^d^, Edgar Hiller ^a^*

^a^ Department of Geochemistry, Faculty of Natural Sciences, Comenius University in Bratislava, Ilkovičova 6, 842 15 Bratislava, Slovak Republic

^b^ SNM-Natural History Museum, Vajanského nábrežie 2, 810 06 Bratislava, Slovak Republic

^c^ The Center of Environmental Services, Ltd., Kutlíkova 17, 852 50 Bratislava, Slovak Republic

^d^ Department of Environmental Geosciences, Faculty of Environmental Sciences, Czech University of Life Sciences Prague, Kamýcká 129, 165 00, Prague – Suchdol, Czech Republic

* Corresponding author: Edgar Hiller, e-mail address: edgar.hiller@uniba.sk, tel.: +421 2 9014 9218. Department of Geochemistry, Faculty of Natural Sciences, Comenius University in Bratislava, Ilkovičova 6, 842 15 Bratislava, Slovak Republic


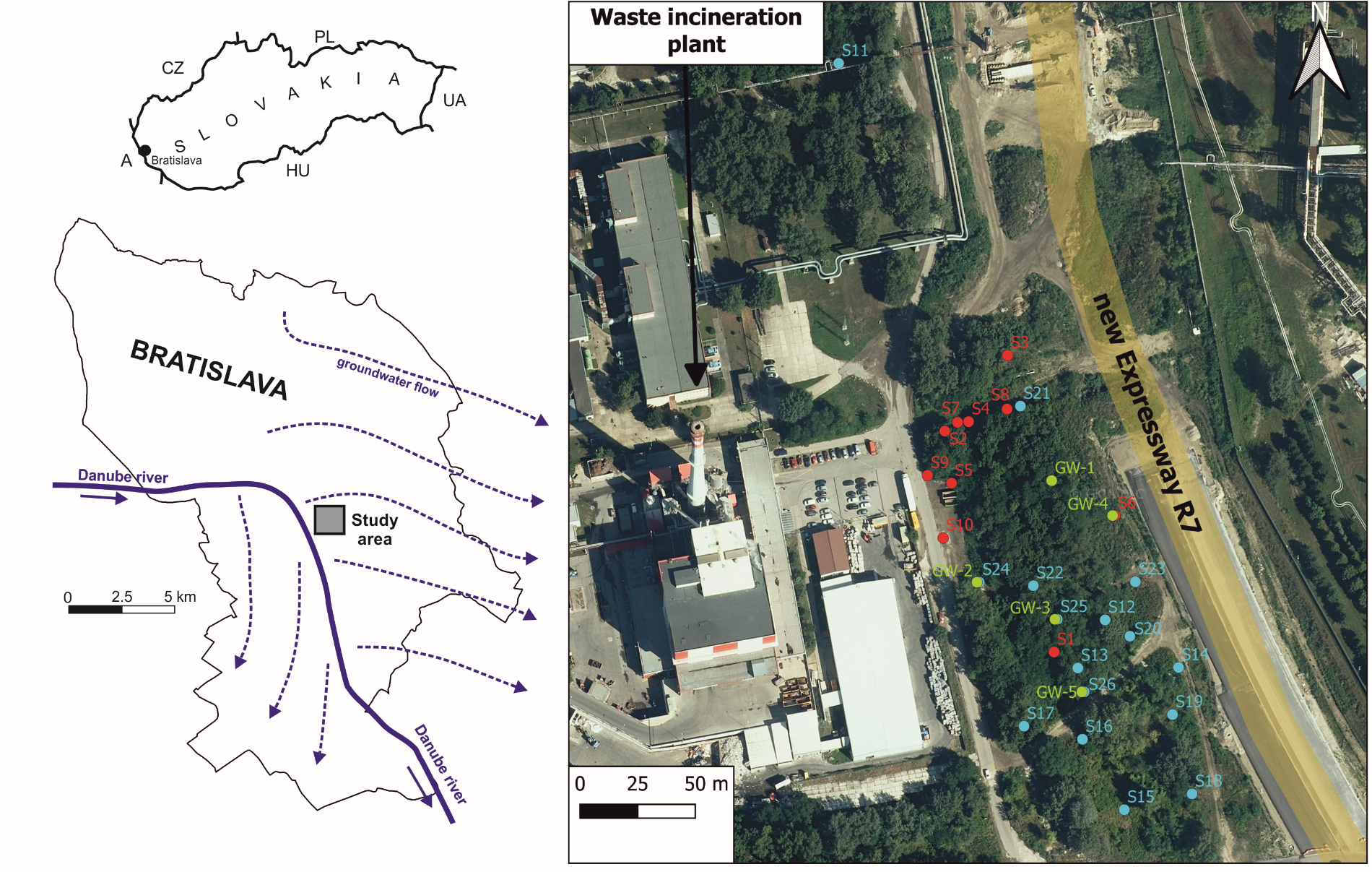


**Figure S1.** Location of the study area and sampling points of soil (S11–S26), groundwater (GW-1–GW-5) and MSWI ashes (S1–S10).

**Table S1.** Background concentrations of target metal(loid)s, indication and intervention criteria for soils of industrial use and corresponding toxicity coefficient values.

|  | mg/kg |  | mg/kg | |  |  |
| --- | --- | --- | --- | --- | --- | --- |
|  | Background value |  | Indication criteria (ID) ^a^ | Intervention criteria (IT) ^a^ |  | Toxicity coefficient (*T*_r_^i^) ^b^ |
| As | 8.03 |  | 65 | 140 |  | 10 |
| Ba | 280 |  | 900 | 2800 |  | NA |
| Cd | 0.40 |  | 10 | 30 |  | 30 |
| Cr | 62.0 |  | 450 | 1000 |  | 2 |
| Cu | 21.0 |  | 500 | 1500 |  | 5 |
| Fe | 21,390 |  | NA ^c^ | NA |  | NA |
| Mn | 442 |  | NA | NA |  | NA |
| Ni | 29.7 |  | 180 | 500 |  | 5 |
| Pb | 16.7 |  | 250 | 800 |  | 5 |
| Sb | 0.63 |  | 25 | 80 |  | 7 |
| Zn | 64.3 |  | 1500 | 5000 |  | 1 |

^a^ according to the Directive of Ministry of Environment of the Slovak Republic No. 1/2015-7 [1]

^b^ according to [2], excepting *T*_r_^i^ value of Sb [3]

^c^ not available

**Supplementary Text S1 – Human health risk assessment**

The investigated area is currently unused. The deposited MSWI ash is found either in the form of piles or at a depth of up to 1.0 m below the surface, and locally, it is incorporated into construction waste of various types. Most of the area is covered with trees reaching a height of up to 10 m. Currently, the construction of administrative buildings is planned in the area, and a new highway has been completed in close proximity to the area. Therefore, construction workers are a risk group who will work here for approximately 2 years for 5 days a week.

The non-carcinogenic health risk was estimated using the hazard quotient (HQ) for each metal(loid) and exposure route, i.e. soil ingestion, inhalation and dermal contact. The sum of HQs of all metal(loid)s for each exposure route represents the hazard index (HI_i_), while the sum of HI_i_s gives the total HI (HI_total_). The HI value below 1.0 means that no non-carcinogenic health risk to exposed population exists. The HI values above 1.0 show that non-carcinogenic health effects may be present [4]. The values of HQ for each exposure route were calculated using the equations:

$HQ=\frac{\mathrm{ADD}}{\mathrm{RfD}} \mathrm{and} \frac{C_{\mathrm{air}}}{\mathrm{RfC}}$ (S1)

$\mathrm{HI}_{i}=\sum_{i=10} \mathrm{HQ}_{i}\mathrm{and}\mathrm{HI}_{\mathrm{total}}=\sum_{i=3} \mathrm{HI}_{i}$ (S2)

where ADD (mg/kg·day) is the average daily dose of a metal(loid) for each exposure pathway, RfD (mg/kg·day) is the respective reference dose, *C*_air_ (mg/m^3^) is the exposure concentration of a metal(loid) in the air and RfC (mg/m^3^) is the respective reference concentration (Table S2).

To assess the carcinogenic risk, cancer risk (Risk) was calculated using the following equation:

$Risk=ADD\times SF and C_{\mathrm{air}}\times IUR$ (S3)

where SF (1/(mg/kg⋅day)) is the slope factor and IUR (1/(μg/m^3^)) is the inhalation unit risk. When the total Risk (Risk_total_ = the sum of Risk values of all metal(loid)s and exposure routes) is below 10^–6^, there is no carcinogenic risk to the exposed population. Among the studied metal(loid)s, As, Cd, Cr, Ni and Pb are considered carcinogenic. These metal(loid)s have specified SF or IUR values. Toxicity values for dermal contact were calculated by multiplying oral RfD values of metal(loid)s or dividing oral SF values with the gastrointestinal absorption factor (GIAF) values shown in Table S2.

The values of ADD and *C*_air_ for three main exposure routes were calculated according to the following equations [5,6]:

$\mathrm{ADD}_{\mathrm{ingestion}}=\frac{C_{\mathrm{total}}\times\mathrm{IngR}_{\mathrm{soil}}\times\mathrm{EF}\times\mathrm{ED}}{\mathrm{BW}\times\mathrm{AT}}\times{10}^{-6}$ (S4)

$C_{\mathrm{air}}=\frac{C_{\mathrm{total}}\times\mathrm{EF}\times\mathrm{ED}}{\mathrm{PEF}\times\mathrm{AT}}$ (S5)

$\mathrm{ADD}_{\mathrm{dermal}}=\frac{C_{\mathrm{total}}\times SA\times SL\times ABF\times EF\times ED}{BW\times AT}\times{10}^{-6}$ (S6)

where *C*_total_ is the total metal(loid) concentration (mg/kg), EF is the exposure frequency (days/year), ED is the exposure duration (years), AT is the averaging time (ED×365 and 70×365 days for non-carcinogenic and carcinogenic risks, respectively), BW is the body weight (kg), IngR_soil_ is the soil ingestion rate (mg/day), PEF is the particle emission factor (m^3^/kg), SA is the exposed skin surface area (cm^2^), SL is the skin adherence factor (mg/cm^2^·day) and ABF is the dimensionless dermal absorption factor. The values of all parameters used in the equations (S4), (S5) and (S6) are listed in Table S3.

**Table S2.** Toxicity characteristics of the studied trace metal(loid)s.

|  | RfD_ingestion_ ^a^ | RfC | Oral SF | IUR | GIAF ^b^ |
| --- | --- | --- | --- | --- | --- |
|  | mg/kg·day | mg/m^3^ | 1/(mg/kg·day) | 1/(μg/m^3^) |  |
| As | 3.0×10^–4^ | 1.5×10^–5^ | 1.5×10^0^ | 4.3×10^–3^ | 0.95 |
| Ba | 2.0×10^–1^ | 5.0×10^–4^ |  |  | 0.07 |
| Cd | 1.0×10^–4^ | 1.0×10^–5^ |  | 1.8×10^–3^ | 0.025 |
| Cr | 1.5×10^0^ | 5.0×10^–3^ |  | 1.2×10^–2^ | 0.013 |
| Cu | 4.0×10^–2^ |  |  |  | 0.57 |
| Mn | 2.4×10^–2^ | 5.0×10^–5^ |  |  | 0.04 |
| Ni | 1.1×10^–2^ | 1.4×10^–5^ |  | 2.4×10^–4^ | 0.04 |
| Pb | 3.6×10^–3^ |  | 8.5×10^–3^ | 1.2×10^–5^ | 0.15 |
| Sb | 4.0×10^–4^ | 3.0×10^–4^ |  |  | 0.15 |
| Zn | 3.0×10^–1^ |  |  |  | 0.20 |

^a^ The values of RfD_ingestion_, RfC, oral SF and IUR were obtained from The Risk Assessment Information System [7] and Integrated Risk Information System [8]

^b^ Gastrointestinal absorption factor values are from The Chemical Properties Database [9]

**Table S3.** Exposure parameters used in human health risk assessment.

| Parameter | Value |
| --- | --- |
| C_total_ = metal(loid) concentration in soil (mg/kg) | 95% UCL |
| IngR_soil_ – soil ingestion rate (mg/day) | 100 |
| EF – exposure frequency (days/year) | 250 |
| ED – exposure duration (years) | 2 |
| AT – averaging time (days) | ED×365 = 730 (non-cancer) |
|  | 70×365 = 25,550 (cancer) |
| BW – body weight (kg) | 80 |
| SA – exposed skin area (cm^2^) | 3527 |
| SL – skin adherence factor (mg/cm^2^) | 0.12 |
| ABF – dermal absorption factor | 0.03 for As; 0.001 for other metal(loid)s |
| PEF – particle emission factor (m^3^/kg) | 1.36×10^9^ |

**Table S4.** Comparison of concentrations of trace metal(loid)s in MSWI ashes stored for a long time in soils in the study area from the city of Bratislava to those in MSWI ashes of different ages from different countries of the world.

| Reference | Country | Age of MSWI ash (years) | Number of samples | As | Ba | Cd | Cr | Cu | Mn | Ni | Pb | Sb | Zn |
| --- | --- | --- | --- | --- | --- | --- | --- | --- | --- | --- | --- | --- | --- |
| *This study* | Slovakia | ~40 | 10 | *22.9* ^a^ | *1969* | *20.6* | *247* | *1697* | *1829* | *132* | *1453* | *59.4* | *6730* |
| Bayuseno and Schmahl [10] | Germany | Fresh | 1 | 21.0 | 3920 | 14.0 | 1158 | 7743 | 1000 | 356 | 1022 | 70.0 | 7732 |
| Huber et al. [11] | Austria | Fresh | 3 | *17.4* | *1130* | *13.4* | *1563* | *9620* | *1603* | *640* | *863* | *13.1* | *5740* |
| Santos et al. [12] | Belgium | Fresh  0.25 | 2  2 | *72.0*  *44.0* | *1513*  *1609* | *5.50*  *85.0* | *219*  *451* | *2623*  *3716* | *1250*  *1650* | *210*  *298* | *720*  *1221* | *196*  *521* | *3022*  *3031* |
| Meima and Comans [13] | The Netherlands | 1.5  12 | 1  1 |  |  | 7.00  14.0 |  | 2190  1700 | 900  770 |  | 1950  1400 |  | 3550  3700 |
| Saffarzadeh et al. [14] | USA | 1  10  13–14  20 | 1  1  1  1 |  |  |  | 358  557  478  314 | 3482  3891  3571  3153 | 1400  2000  2600  1200 | 357  242  606  289 | 3812  3977  3430  3016 |  | 10,064  8552  7811  5702 |
| Polettini and Pomi [15] | Italy | Fresh | 1 |  |  | <8.0 | 168 | 1084 | 72 | 332 | 80.0 |  | 201 |
| Wei et al. [16] | Japan | Fresh | 4 | *<1.0* | *952* | *<1.0* | *339* | *2200* | *875* | *109* | *903* | *4.00* | *3210* |
| Gupta et al. [17] | India | Fresh | 6 | *6.00* | *469* | *4.00* | *296* | *382* | *417* | *68.0* | *142* | *10.0* | *976* |
| Yao et al. [18] | China | Fresh | 6 | *85.3* |  | *4.97* | *349* | *562* | *787* | *29.7* | *361* |  | *1567* |
| Blanc et al. [19] | France | ~0.75 | 1 | 15.1 | 916 |  | 479 | 1266 |  | 217 | 737 | 67.7 | 2448 |
| van der Sloot et al. [20] | The Netherlands | Fresh | NA ^b^ | *6.60* |  | *4.00* | *117* | *2200* |  |  | *1270* | *34.0* | *1976* |
| Nguyen et al. [21] | Vietnam | Fresh | 9 | *13.2* |  | *9.63* | *143* | *975* |  | *69.5* | *91.1* |  | *1628* |

^a^ The mean value is shown by italics

^b^ Not available information


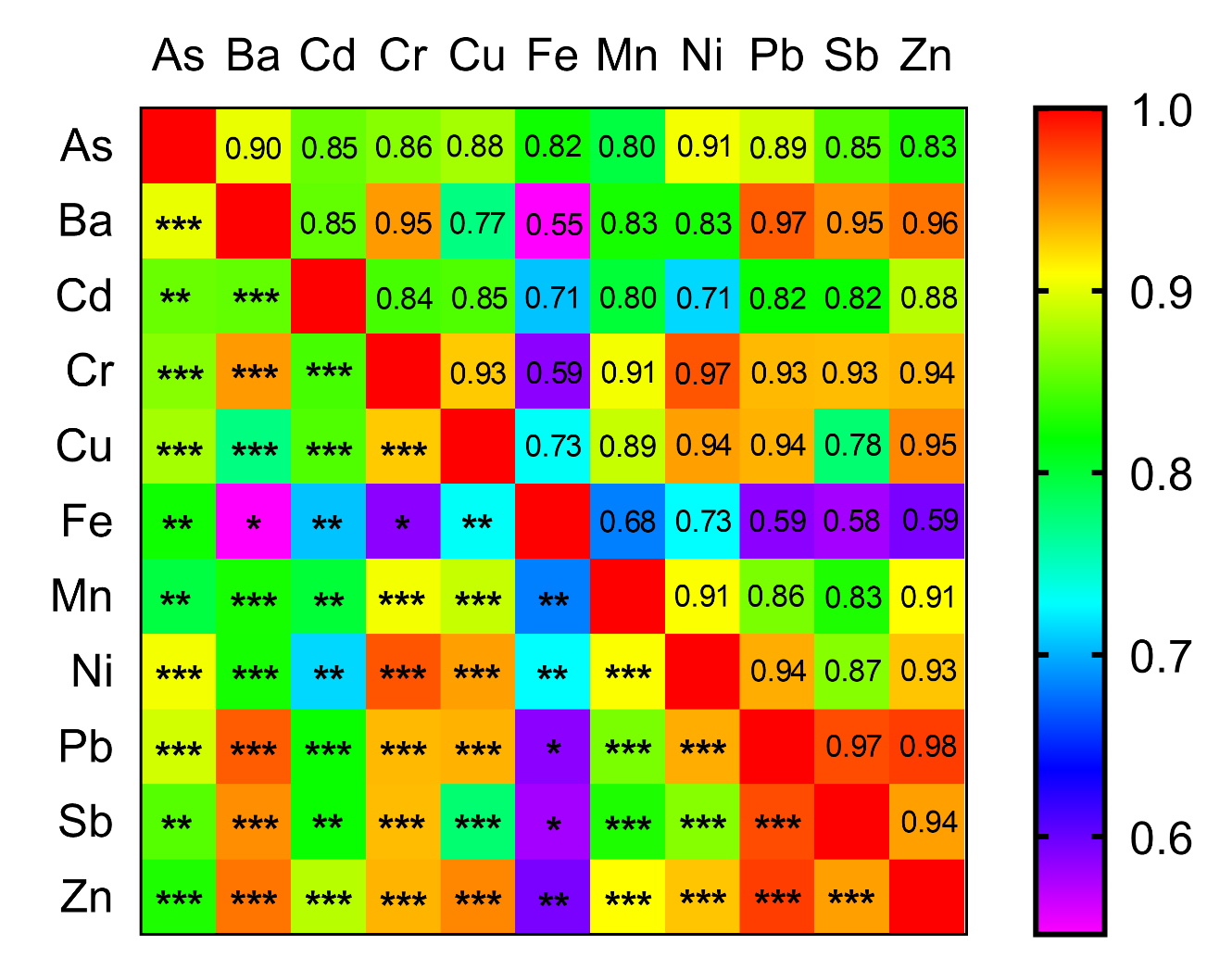


**Figure S2.** Spearman correlation matrix showing significant positive relationships between individual metal(loid) pairs in soils and MSWI ash residues. Symbols “*******”, “******” and “*****” indicate the significance level at *p* <0.001, *p* <0.01 and *p* <0.05, respectively.


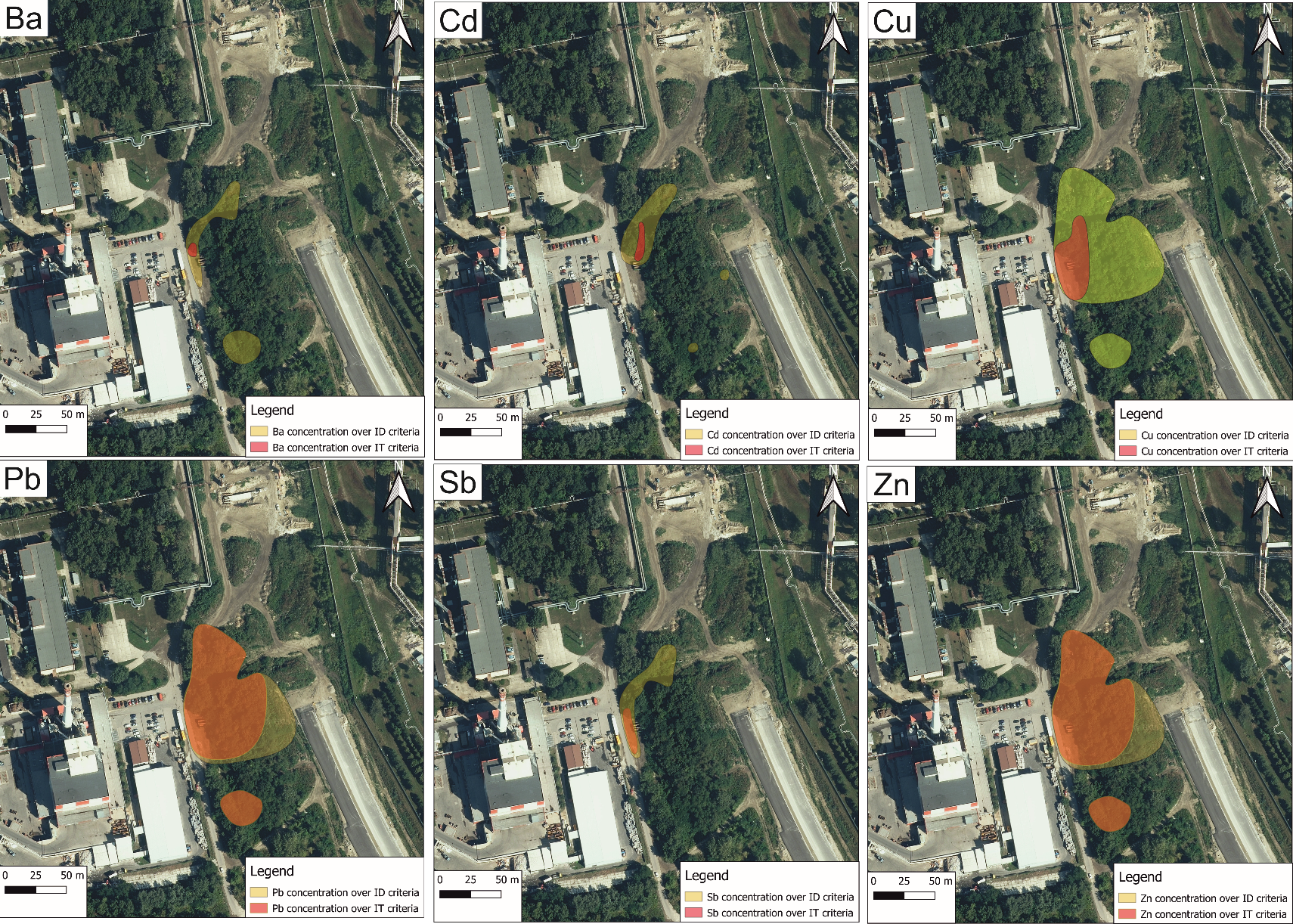


**Figure S3.** Spatial distribution maps showing sites where total concentrations of Ba, Cd, Cu, Pb, Sb and Zn exceeded their respective indication and intervention criteria (ID and IT, respectively) defined for soils of industrial use. The values of ID and IT (all in mg/kg) are 900 and 2800 for Ba, 10.0 and 30.0 for Cd, 500 and 1500 for Cu, 250 and 800 for Pb, 25 and 80 for Sb, and 1500 and 5000 for Zn, respectively [1].

**Table S5.** Dissolved concentrations of major metals and anions in the leachates (mg/L) after extraction of MSWI ash samples with low molecular weight organic acids and dissolved fractions, i.e. % of the total concentration in the sample (shown in parentheses).

|  |  | Samples |  |  |  |  |  |
| --- | --- | --- | --- | --- | --- | --- | --- |
|  | Treatment | S7 | S8 | S9a | S9b | S10a | S10b |
| Al | 1.0 mM CA ^a^ | 2.66(0.07) | 2.53(0.06) | 2.06(0.04) | 2.83(0.06) | 2.28(0.04) | 3.44(0.06) |
|  | 0.1 mM CA | 0.13(0.00) | 0.10(0.00) | 0.11(0.00) | 0.11(0.00) | 0.16(0.00) | 0.16(0.00) |
|  | 1.0 mM OA ^a^ | 0.19(0.01) | 0.07(0.00) | 0.07(0.00) | 0.09(0.00) | 0.11(0.00) | 0.12(0.00) |
|  | 0.1 mM OA | 0.28(0.01) | 0.11(0.00) | 0.09(0.00) | 0.15(0.00) | 0.18(0.00) | 0.18(0.00) |
| Ca | 1.0 mM CA | 98.3(1.05) | 95.5(1.41) | 103(1.14) | 228(2.56) | 73.0(0.88) | 146(1.74) |
|  | 1.0 mM CA | 42.0(0.45) | 35.4(0.52) | 52.9(0.58) | 187(2.10) | 24.1(0.29) | 107(1.27) |
|  | 1.0 mM OA | 56.1(0.60) | 48.1(0.71) | 69.6(0.77) | 202(2.27) | 32.7(0.39) | 100(1.19) |
|  | 0.1 mM OA | 40.0(0.43) | 32.4(0.48) | 54.3(0.60) | 141(1.59) | 21.7(0.26) | 95.7(1.14) |
| K | 1.0 mM CA | 23.1(1.78) | 8.74(0.82) | 9.82(0.84) | 8.64(0.74) | 9.13(0.79) | 20.5(1.69) |
|  | 0.1 mM CA | 21.0(1.61) | 8.58(0.81) | 9.43(0.80) | 9.41(0.80) | 8.31(0.72) | 20.4(1.69) |
|  | 1.0 mM OA | 23.0(1.77) | 8.36(0.79) | 10.9(0.93) | 9.30(0.79) | 8.76(0.76) | 19.7(1.63) |
|  | 0.1 mM OA | 21.0(1.61) | 8.01(0.76) | 9.59(0.82) | 7.76(0.66) | 7.32(0.63) | 19.4(1.60) |
| Mg | 1.0 mM CA | 10.6(1.08) | 7.60(0.61) | 9.80(1.07) | 7.80(0.87) | 9.55(0.91) | 23.0(2.48) |
|  | 0.1 mM CA | 6.36(0.65) | 4.68(0.37) | 7.46(0.81) | 7.02(0.78) | 5.34(0.51) | 20.8(2.24) |
|  | 1.0 mM OA | 8.64(0.88) | 5.96(0.47) | 9.99(1.09) | 8.01(0.89) | 7.29(0.69) | 20.6(2.22) |
|  | 0.1 mM OA | 6.18(0.63) | 4.15(0.33) | 7.80(0.85) | 5.56(0.62) | 4.47(0.43) | 18.6(2.00) |
| Na | 1.0 mM CA | 7.60(NA) ^b^ | 5.20(NA) | 36.3(NA) | 26.3(NA) | 38.1(NA) | 164(NA) |
|  | 0.1 mM CA | 6.87(NA) | 4.96(NA) | 37.8(NA) | 28.5(NA) | 38.1(NA) | 168(NA) |
|  | 1.0 mM OA | 7.67(NA) | 5.10(NA) | 42.8(NA) | 28.1(NA) | 38.2(NA) | 155(NA) |
|  | 0.1 mM OA | 7.27(NA) | 5.00(NA) | 38.1(NA) | 26.0(NA) | 35.4(NA) | 160(NA) |
| Cl^–^ | 1.0 mM CA | 6.80(6.73) | <5.0 | 30.5(19.5) | 17.8(15.4) | 16.3(12.7) | **197**(25.5) ^c^ |
|  | 0.1 mM CA | 6.30(6.23) | <5.0 | 30.0(19.2) | 17.5(15.2) | 16.6(12.9) | **193**(25.0) |
|  | 1.0 mM OA | 6.40(6.33) | <5.0 | 33.2(21.3) | 18.1(15.7) | 16.0(12.4) | **185**(23.9) |
|  | 0.1 mM OA | 6.00(5.93) | <5.0 | 30.0(19.2) | 17.9(15.5) | 15.7(12.2) | **190**(24.6) |
| SO_4_^2–^ | 1.0 mM CA | 10.0(3.91) | 28.3(13.7) | **123**(38.4) | **423**(69.9) | 53.4(18.6) | **217**(42.3) |
|  | 0.1 mM CA | <5.0 | 34.8(16.9) | **128**(40.0) | **443**(73.2) | 60.2(21.0) | **229**(44.6) |
|  | 1.0 mM OA | <5.0 | 37.7(18.3) | **138**(43.1) | **452**(74.7) | 59.2(20.6) | **216**(42.1) |
|  | 0.1 mM OA | <5.0 | 39.8(19.3) | **132**(41.2) | **348**(57.5) | 54.2(18.9) | **206**(40.2) |
| HCO_3_^–^ | 1.0 mM CA | 406 | 341 | 284 | 272 | 208 | 439 |
|  | 0.1 mM CA | 201 | 115 | 100 | 103 | 111 | 286 |
|  | 1.0 mM OA | 262 | 156 | 161 | 140 | 149 | 258 |
|  | 0.1 mM OA | 198 | 95.6 | 102 | 66.7 | 99.4 | 252 |

^a^ CA = citric acid solution, OA = oxalic acid solution

^b^ Not available because the total Na concentration was not determined

^c^ Concentrations of anions (SO_4_^2–^ and Cl^–^) in the leachates marked in bold type exceeded the criteria for inert waste (100 mg/L for SO_4_^2–^ and 80 mg/L for Cl^–^) according to the legislation of the European Union [22]

**Table S6.** The main dissolved species of major metals in the leachates from weathered MSWI ashes. Values are expressed in % and represent arithmetic mean ± standard deviation from six samples. Only species with fractions above 1% are shown.

| Species | 1 mM citric acid | 0.1 mM citric acid | Species | 1 mM oxalic acid | 0.1 mM oxalic acid |
| --- | --- | --- | --- | --- | --- |
| Al(OH)_3_^0^ | 1.40±0.30 | 1.65±0.67 | Al(OH)_3_^0^ | <1 | 1.26±0.29 |
| Al(OH)_4_^–^ | 98.6±0.29 | 98.3±0.70 | Al(OH)_4_^–^ | 57.8±10.5 | 91.6±3.32 |
|  |  |  | Al(OH)_2_-oxalate^–^ | 40.5±10.0 | 7.10±3.04 |
| Ca^2+^ | 64.9±7.34 | 74.6±12.3 | Ca^2+^ | 68.2±8.74 | 76.6±11.8 |
| CaSO_4_^0^ | 15.5±12.5 | 23.4±14.3 | CaSO_4_^0^ | 21.8±13.8 | 23.2±13.4 |
| CaHCO_3_^+^ | 2.60±0.97 | 1.49±0.79 | CaHCO_3_^+^ | 1.67±0.76 | 1.40±0.82 |
| CaCO_3_^0^ | 1.81±0.57 | <1 | CaCO_3_^0^ | 1.26±0.72 | 1.11±0.65 |
| Ca-citrate^–^ | 15.0±5.02 | 3.30±1.88 | Ca-oxalate^0^ | 10.6±4.35 | 1.47±0.66 |
| Fe(OH)_2_^+^ | 79.0±6.93 | 81.0±9.13 | Fe(OH)_2_^+^ | 77.5±8.47 | 77.3±8.43 |
| Fe(OH)_3_^0^ | 5.10±0.85 | 4.80±1.22 | Fe(OH)_3_^0^ | 5.02±1.19 | 5.35±1.00 |
| Fe(OH)_4_^–^ | 15.7±6.10 | 14.2±7.94 | Fe(OH)_4_^–^ | 15.7±7.89 | 17.3±7.43 |
|  |  |  | Fe-(oxalate)_3_^3–^ | 1.61±0.70 | <1 |
| K^+^ | 98.5±1.41 | 98.4±1.51 | K^+^ | 98.3±1.49 | 98.5±1.28 |
| KSO_4_^–^ | 1.42±1.39 | 1.87±1.49 | KSO_4_^–^ | 1.87±1.49 | 1.72±1.26 |
| Mg^2+^ | 67.4±6.16 | 77.9±10.6 | Mg^2+^ | 60.1±4.11 | 78.0±9.43 |
| MgSO_4_^0^ | 13.0±10.7 | 19.9±12.4 | MgSO_4_^0^ | 16.1±11.1 | 19.2±11.4 |
| MgHCO_3_^+^ | 2.17±0.77 | 1.25±0.64 | MgHCO_3_^+^ | 1.18±0.49 | 1.14±0.65 |
| Mg-citrate^–^ | 16.2±5.17 | 3.58±2.00 | Mg-oxalate^0^ | 24.5±8.51 | 4.00±1.70 |
| Na^+^ | 98.6±1.08 | 98.6±1.18 | Na^+^ | 98.5±1.14 | 98.7±0.99 |
| NaSO_4_^–^ | 1.10±1.09 | 1.46±1.17 | NaSO_4_^–^ | 1.46±1.17 | 1.34±0.99 |


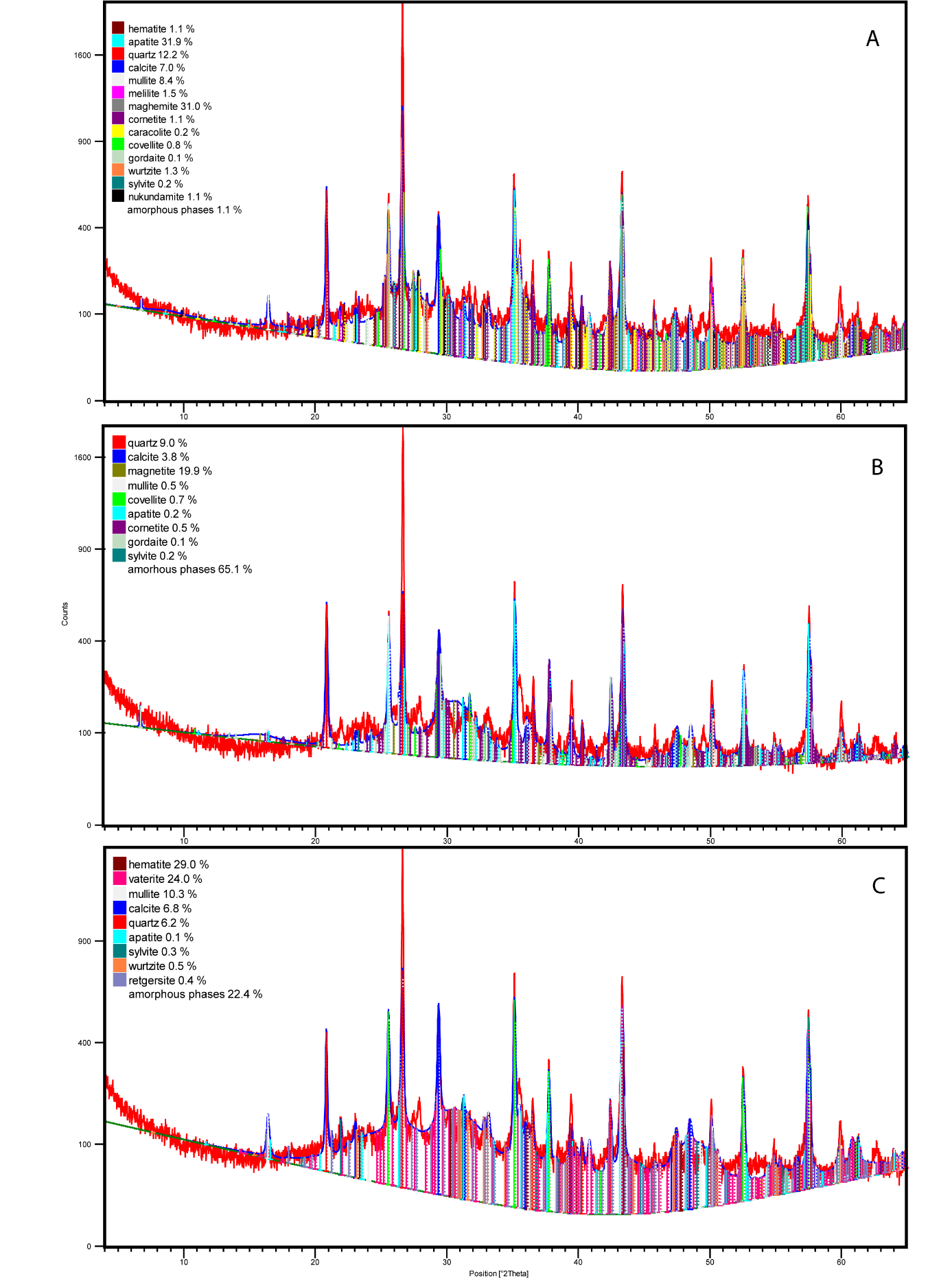


**Figure S4.** X-ray patterns of weathered MSWI ash (sample S9a): **a)** original ash before extraction with organic acids, **b)** after extraction with an aqueous solution of 1 mM citric acid and **c)** after extraction with an aqueous solution of 1 mM oxalic acid.

**Table S7.** The range of saturation index (SI) values of selected minerals in leachates from the extraction of six MSWI ash samples with citric and oxalic acid solutions.

| Mineral name | Formula | 1 mM citric acid | 0.1 mM citric acid | 1 mM oxalic acid | 0.1 mM oxalic acid |
| --- | --- | --- | --- | --- | --- |
| Otavite | CdCO_3_ | –0.62 – +0.16 ^a^ | –9.83 – –9.13 | –9.81 – –9.12 | –9.92 – –9.06 |
| Azurite | Cu_3_(CO_3_)_2_(OH)_2_ | +0.36 – +2.17 | –2.44 – +0.28 | –5.62 – –1.09 | –3.06 – +1.00 |
| Brochantite | Cu_4_(SO_4_)(OH)_6_ | –2.85 – +1.64 | –14.7 – –2.89 | –16.7 – –6.88 | –13.6 – –2.90 |
| Malachite | Cu_2_(CO_3_)(OH)_2_ | +0.97 – +2.24 | –0.75 – +0.95 | –2.82 – +0.11 | –1.10 – +1.52 |
| Tenorite (amorphous) | CuO | –0.45 – +0.35 | –1.11 – –0.41 | –2.13 – –0.73 | –1.18 – +0.02 |
| Ferrihydrite | 5Fe_2_O_3_·9H_2_O | +5.08 – +5.49 | +3.32 – +4.36 | +3.31 – +4.88 | +3.36 – +5.10 |
| Goethite | α-FeO(OH) | +7.79 – +8.20 | +6.03 – +7.07 | +6.02 – +7.59 | +6.07 – +7.81 |
| Hematite | Fe_2_O_3_ | +18.0 – +18.8 | +14.5 – +16.5 | +14.5 – +17.6 | +14.5 – +18.0 |
| Maghemite | γ-Fe_2_O_3_ | +10.2 – +11.0 | +6.65 – +8.74 | +6.64 – +9.78 | +6.73 – +10.2 |
| Gibbsite | Al(OH)_3_ | +2.88 – +3.22 | +1.64 – +2.07 | +1.22 – +1.73 | +1.58 – +2.10 |
| Smithsonite | ZnCO_3_ | +0.20 – +0.73 | –1.16 – –0.22 | –24.8 – –1.36 | –1.40 – +0.15 |
| Zincite | (Zn,Mn)O | –1.11 – –0.67 | –2.18 – –1.67 | –2.56 – –1.56 | –2.12 – –1.01 |
| Rhodochrosite | MnCO_3_ | –0.32 – +0.36 | –2.01 – –0.84 | –1.43 – –0.66 | –1.89 – –0.78 |
| Barite | BaSO_4_ | +0.11 – +0.84 | +0.45 – +0.90 | +0.51 – +0.90 | +0.51 – +0.97 |
| Ca oxalate trihydrate | CaC_2_O_4_∙3H_2_O | – | – | +1.21 – +1.52 | +0.20 – +0.40 |
| Calcite | CaCO_3_ | +0.77 – +1.07 | +0.10 – +0.94 | +0.25 – +0.78 | +0.22 – +0.83 |
| Vaterite | CaCO_3_ | +0.21 – +0.50 | –0.47 – +0.37 | –0.31 – +0.21 | –0.47 – +0.26 |
| Gypsum | CaSO_4_⋅2H_2_O | –2.06 – –0.44 | –14.7 – –0.47 | –14.7 – –0.45 | –14.7 – –0.52 |
| Magnesite | MgCO_3_ | –1.33 – –0.51 | –1.83 – –0.55 | –1.72 – –0.91 | –2.03 – –0.66 |
| K-jarosite | KFe^3+^_3_(SO_4_)_2_(OH)_6_ | +2.13 – +3.59 | –25.5 – –1.29 | –24.8 – –1.36 | –24.1 – –6.42 |

^a^ SI >0 = the solution is supersaturated with a mineral, SI <0 = the leachate is unsaturated with a mineral, the SI interval <–1.0; +1.0> indicates a saturation state

**Table S8.** Data on metal(loid) concentrations (mg/kg) in soils near MSW incinerators and contaminated by MSWI ashes from different countries and their comparison to those from the study area. Concentrations are shown as arithmetic mean±standard deviation and range (italics numbers).

| Reference | Type of samples | Country/City | Grain size (mm) | As | Cd | Cr | Cu | Mn | Ni | Pb | Sb | Zn |
| --- | --- | --- | --- | --- | --- | --- | --- | --- | --- | --- | --- | --- |
| ***This study*** | ~40 year old ash | Slovakia/Bratislava | <2.0 | 22.9±5.5  *15.6–27.0* | 20.6±6.6  *11.8–30.4* | 247±199  *42.1–577* | 1697±1025  *521–4490* | 1824±436  *1104–5212* | 132±87.5  *42.5–312* | 1453±505  *605–2320* | 59.4±28.8  *21.2–99.0* | 6731±2515  *2670–10950* |
|  | Surrounding soil |  |  | 4.0±2.1  *1.7–8.6* | <0.4 | 15.3±4.3  *8.1–22.2* | 11.4±6.2  *3.6–19.8* | 248±95.4  *130–367* | 14.3±5.4  *6.7–23.0* | 10.1±6.2  2.8–32.2 | <0.5 | 44.3±34.1  *14.5–144* |
| Xiong et al. [23] | ~25 year old ash | Japan | <2.0 |  | 27.0±25.9  *5.2–75.6* | 272±134  *84.6–457* | 1756±1136  *376–3520* | 1150±362  *600–1500* | 157±92.8  *47.6–284* | 1504±1007  *193–3270* |  | 3697±2947  *646–8730* |
|  | Soil covering the ash |  |  |  | 2.4±1.6  *1.0–4.67* | 67.0±10.4  *56.4–83.1* | 48.6±20.2  *27.4–89.3* | 825±205  *600–1100* | 59.1±17.1  *31.9–88.2* | 37.7±21.3  *10.9–75.3* |  | 132±63.4  *60.8–231* |
| Rigo et al. [24] | ~35 year old ash | Lagoon of Venice | <2.0 | 23.8 | 3.4 | 100 | 1800 | 1138 | 80.0 | 2000 |  | 3225 |
| Gwenzi et al. [25] | Old ash | Zimbabwe/Harare | <2.0 |  | 3.5 |  | 185 | 470 | 19.5 | 90 |  | 510 |
|  | Soil beneath dump |  |  |  | 1.6 |  | 180 | 800 | 18.8 | 22 |  | 100 |
| Adama et al. [26] | Relatively fresh ash | Ghana | <2.0 |  | 7.5±2.7 | 99.3±3.0 |  |  |  | 144±39.5 |  | 16418±195 |
|  | Uncontaminated soil |  |  |  | 0.5±0.0 | 2.32±0.1 |  |  |  | 4.9±0.1 |  | 15.0±0.5 |
| Mouedhen et al. [27] | ~65 year old ash + soil | Canada/Québec | <2.0 |  |  |  | 654±313  *183–1120* |  |  | 1867±779  *942–3240* |  | 1573±791  *514–3230* |
| Jobin et al. [28] | ~65 year old ash + soil | Canada/Québec | <2.0 | 44.0 |  |  | 1774 |  |  | 5025 |  | 3544 |
| Rimmer et al. [29] | Soil near incinerator | UK/Newcastle upon Tyne | <2.0 | 20  *5.0–279* | 0.65  *0.01–7.0* | 55  *23–230* | 233  *20–12107* |  | 30  *11–165* | 350  *40–4134* |  | 419  *75–4625* |
| Rovira et al. [30] | Soil near incinerator | Spain/Mataró | <2.0 | 0.9 | 0.05 | 10.3 | 10.4 | 350 | 2.8 | 9.4 | BDL | 56.1 |
| Rovira et al. [31] | Soil near incinerator | Catalonia/Girona | <2.0 | 4.6±2.4 | 0.2±0.2 | 9.3±5.8 | 25.3±46.3 | 344±156 | 11.4±6.5 | 18.2±20.5 | 0.2±0.3 |  |
| Bretzel and Calderisi [32] | Soil near incinerator | Italy/Pisa | <2.0 | 5.9±1.9  *2.6–12.6* | 0.5±0.3  *0.02–1.3* | 84.7±24.3  *14.9–135* | 68.7±43.3  *0.15–271* |  |  | 39.3±34.8  *14.6–207* |  | 127±135  *48.4–1286* |
| Meneses et al. [33] | Soil near incinerator | Spain/Barcelona | <2.0 | 9.7±3.2 | 0.4±0.3 | 17.7±5.8 |  | 423±76.3 | 17.4±8.8 | 54.2±47.4 |  | 97.8±61.1 |
| Vilavert et al. [34] | Soil near incinerator | Spain/Taragona | <2.0 | 5.1±1.5 | 0.2±0.06 | 17.8±6.1 | 28.2±24.2 | 236±122 | 9.16±2.9 | 39.5±30.0 | 0.2±0.1 |  |
| Ma et al. [35] | Soil near incinerator | North China | <0.15 | 31.2±18.6 | 0.4±0.1 | 76.4±25.0 | 30.5±7.1 |  | 31.8±6.8 | 26.7±9.0 |  | 120±42.7 |
| Bo et al. [36] | Soil near incinerator | Southwest China | <0.15 | 6.9  *2.1–15.3* | 0.3  *0.1–0.5* | 81.2  *59.1–97.3* | 34.1  *13.6–61.5* | 673  *180–1203* | 40.5  *23.3–56.1* | 25.7  *18.6–40.8* | 1.0  *0.4–1.9* | 194  *76.0–1390* |
| Li et al. [37] | Soil near incinerator | South China/Guangzhou | <2.0 |  |  | 285  *215–358* | 13.5  *7.1–21.4* |  | 10.1  *8.0–15.0* | 15.0  *11.9–21.8* |  |  |
| Li et al. [38] | Soil near incinerator | China/Shenzhen | <2.0 |  | 0.3±0.2 | 37.1±24.3 |  | 322±359 |  | 58.8±33.5 |  |  |

*Continuing* **Table S8**

| Reference | Type of samples | Country/City | Grain size (mm) | As | Cd | Cr | Cu | Mn | Ni | Pb | Sb | Zn |
| --- | --- | --- | --- | --- | --- | --- | --- | --- | --- | --- | --- | --- |
| Richardson [39] | Soil near incinerator | USA/Poughkeepsie | <2.0 | 4.4±0.4 | 0.5±0.0 |  | 11±1 | 2760±513 | 34±4 | 106±16 |  | 187±27 |
|  |  | USA/Hartford | <2.0 | 1.7±1.1 | 0.3±0.1 |  | 13±3 | 394±177 | 11±4 | 67±26 |  | 87±25 |
|  |  | USA/Springfield | <2.0 | 1.4±0.2 | 0.1±0.0 |  | 10±2 | 189±43 | 6±1 | 104±14 |  | 52±5 |
| Li et al. [40] | Soil near incinerator | Southeast China/Shanghai | <2.0 |  | 0.2±0.08  *0.08–0.6* | 77.2±12.5  *54.5–134* | 31.2±6.1  *19.1–65.9* |  | 29.7±5.4  *22.3–45.1* | 32.2±9.5  *20.4–73.3* | 1.2±0.3  *0.6–3.3* | 107±14.6  *80.8–182* |
| Li et al. [41] | Soil near incinerator | Northeast China | <2.0 | 16.0±11.9  *6.8–67.7* | 0.7±2.0  *0.03–11.9* | 84.9±56.8  *31.6–319* | 50.8±111  *7.9–534* | 613±155  *285–1192* | 30.2±8.3  *12.3–55.8* | 53.4±90.6  *20.1–480* |  | 177±426  *26.1–1997* |

**References**

[1] Anon, 2015. Directive of Ministry of Environment of the Slovak Republic of January 28, 2015 No. 1/2015 – 7 to develop a risk analysis of the contaminated area (in Slovak). https://www.minzp.sk/files/sekcia-geologie-prirodnych-zdrojov/ar_smernica_final.pdf (accessed 21 April 2022).

[2] Håkanson, L., 1980. An ecological risk index for aquatic pollution control: a sedimentological approach. Water Res. 14, 975–1001. https://doi.org/10.1016/0043-1354(80)90143-8.

[3] Wang, N., Wang, A., Kong, L., He, M., 2018. Calculation and application of Sb toxicity coefficient for potential ecological risk assessment. Sci. Total Environ. 610–611, 167–174. https://doi.org/10.1016/j.scitotenv.2017.07.268.

[4] USEPA, 1989. Risk assessment guidance for superfund (RAGS), Volume I, Human health evaluation manual (Part A). U.S. Environmental Protection Agency, Washington, DC, Office of Emergency and Remedial Response, EPA/540/1-89/002. https://www.epa.gov/risk/risk-assessment-guidance-superfund-rags-part (accessed 18 September 2022).

[5] Izquierdo, M., De Miguel, E., Ortega, M.F., Mingot, J., 2015. Bioaccessibility of metals and human health risk assessment in community urban gardens. Chemosphere. 135, 312–318. https://doi.org/10.1016/j.chemosphere.2015.04.079.

[6] USEPA, 2022a. EPA ExpoBox (A Toolbox for Exposure Assessors). United States Environmental Protection Agency. https://www.epa.gov/expobox (accessed 10 May 2022).

[7] US DoE, 2022. The Risk Assessment Information System. US Department of Energy, Washington DC. https://rais.ornl.gov/ (accessed 10 May 2022).

[8] USEPA, 2022b. Integrated Risk Information System. United States Environmental Protection Agency. https://www.epa.gov/iris (accessed 10 May 2022).

[9] GSI, 2019. Chemical properties database, GSI Environmental Inc. http://www.gsi-net.com/en/publications/gsi-chemical-database.html. (accessed 17 July 2019).

[10] Bayuseno, A.P., Schmahl, W.W., 2010. Understanding the chemical and mineralogical properties of the inorganic portion of MSWI bottom ash. Waste Manag. 30, 1509–1520. https://doi.org/10.1016/j.wasman.2010.03.010.

[11] Huber, F., Blasenbauer, D., Aschenbrenner, P., Fellner, J., 2019. Chemical composition and leachability of differently sized material fractions of municipal solid waste incineration bottom ash. Waste Manag. 95, 593–603. https://doi.org/10.1016/j.wasman.2019.06.047.

[12] Santos, R.M., Mertens, G., Salman, M., Cizer, Ö., Van Gerven, T., 2013. Comparative study of ageing, heat treatment and accelerated carbonation for stabilization of municipal solid waste incineration bottom ash in view of reducing regulated heavy metal/metalloid leaching. J. Environ. Manag. 128, 807–821. https://doi.org/10.1016/j.jenvman.2013.06.033.

[13] Meima, J.A., Comans, R.N.J., 1999. The leaching of trace elements from municipal solid waste incinerator bottom ash at different stages of weathering. Appl. Geochem. 14, 159–171. https://doi.org/10.1016/S0883-2927(98)00047-X.

[14] Saffarzadeh, A., Shimaoka, T., Wei, Y., Gardner, K.H., Musselman, C.H., 2011. Impacts of natural weathering on the transformation/neoformation processes in landfilled MSWI bottom ash? A geoenvironmental perspective. Waste Manag. 31, 2440–2454. https://doi.org/10.1016/j.wasman.2011.07.017.

[15] Polettini, A., Pomi, R., 2004. The leaching behavior of incinerator bottom ash as affected by accelerated ageing. J. Hazard. Mater. 113, 209–215. https://doi.org/10.1016/j.jhazmat.2004.06.009.

[16] Wei, Y., Shimaoka, T., Saffarzadeh, A., Takahashi, F., 2011. Mineralogical characterization of municipal solid waste incineration bottom ash with an emphasis on heavy metal-bearing phases. J. Hazard. Mater. 187, 534–543. https://doi.org/10.1016/j.jhazmat.2011.01.070.

[17] Gupta, G., Datta, M., Ramana, G. V., Alappat, B. J., Bishnoi, S., 2021. Contaminants of concern (CoCs) pivotal in assessing the fate of MSW incineration bottom ash (MIBA): First results from India and analogy between several countries. Waste Manag. 135, 167–181. https://doi.org/10.1016/j.wasman.2021.08.036.

[18] Yao, J., Li, W.-B., Kong, Q.-N., Wu, Y.-Y., He, R., Shen, D.-S., 2010. Content, mobility and transfer behavior of heavy metals in MSWI bottom ash in Zhejiang province, China. Fuel. 89, 616–622. https://doi.org/10.1016/j.fuel.2009.06.016.

[19] Blanc, D., Gonzalez, L., Lupsea‑Toader, M., de Brauer, C., 2018. Mineralogical evolution and leaching behaviour of a heap of bottom ash as a function of time: Influence on its valorization. Waste Biomass Valoriz. 9, 2517–2527. https://doi.org/10.1007/s12649-018-0444-1.

[20] van der Sloot, H.A., Kosson, D.S., Hjelmar, O., 2001. Characteristics, treatment and utilization of residues from municipal waste incineration. Waste Manag. 21, 753–765. https://doi.org/10.1016/S0956-053X(01)00009-5.

[21] Nguyen, T.H., Pham, Q.V., Nguyen, T.P.M., Vu, V.T., Do, T.H., Hoang, M.T., Thi, N.T.T., Minh, T.B., 2022. Distribution characteristics and ecological risks of heavy metals in bottom ash, fly ash, and particulate matter released from municipal solid waste incinerators in northern Vietnam. Environ. Geochem. Health (In Press). https://doi.org/10.1007/s10653-022-01335-4.

[22] EU Council, 2003. Council decision of 19 December 2002 establishing criteria and procedures for the acceptance of waste at landﬁlls pursuant to Article 16 of and Annex II to Directive 1999/31/EC. The Council of the European Union. Off. J. Eur. Commun. L11, 27–49. https://eur-lex.europa.eu/legal-content/EN/TXT/PDF/?uri=CELEX:32003D0033&from=GA (accessed 14 June 2022).

[23] Xiong, Y., Takaoka, M., Sano, A., Kusakabe, T., Yang, J., Shiota, K., Fujimori, T., Oshita, K., 2019. Distribution and characteristics of heavy metals in a first-generation monofill site for incinerator residue. J. Hazard. Mater. 373, 763–772. https://doi.org/10.1016/j.jhazmat.2019.04.019.

[24] Rigo, C., Zamengo, L., Rampazzo, G., Argese, E., 2009. Characterization of a former dump site in the Lagoon of Venice contaminated by municipal solid waste incinerator bottom ash, and estimation of possible environmental risk. Chemosphere. 77, 510–517. https://doi.org/10.1016/j.chemosphere.2009.07.046.

[25] Gwenzi, W., Gora, D., Chaukura, N., Tauro, T., 2016. Potential for leaching of heavy metals in open-burning bottom ash and soil from a non-engineered solid waste landfill. Chemosphere. 147, 144–154. https://doi.org/10.1016/j.chemosphere.2015.12.102.

[26] Adama, M., Esena, R., Fosu-Mensah, B., Yirenya-Tawiah, D., 2016. Heavy metal contamination of soils around a hospital waste incinerator bottom ash dumps site. J. Environ. Public Health. 2016, 8926453. https://doi.org/10.1155/2016/8926453.

[27] Mouedhen, I., Coudert, L., Blais, J.F., Mercier, G., 2019. Prediction of physical separation of metals from soils contaminated with municipal solid waste ashes and metallurgical residues. Waste Manage. 93, 138–152. https://doi.org/10.1016/j.wasman.2019.05.031.

[28] Jobin, P., Mercier, G., Blais, J.F., 2016. Magnetic and density characteristics of a heavily polluted soil with municipal solid waste incinerator residues: Significance for remediation strategies. Int. J. Miner. Process. 149, 119–126. https://doi.org/10.1016/j.minpro.2016.02.010.

[29] Rimmer, D.L., Vizard, C.G., Pless-Mulloli, T., Singleton, I., Air, V.S., Keatinge, Z.A.F., 2006. Metal contamination of urban soils in the vicinity of a municipal waste incinerator: One source among many. Sci. Total Environ. 356, 207–216. https://doi.org/10.1016/j.scitotenv.2005.04.037.

[30] Rovira, J., Vilavert, L., Nadal, M., Schuhmacher, M., Domingo, J.L., 2015. Temporal trends in the levels of metals, PCDD/Fs and PCBs in the vicinity of a municipal solid waste incinerator. Preliminary assessment of human health risks. Waste Manage. 43, 168–175. https://doi.org/10.1016/j.wasman.2015.05.039.

[31] Rovira, J., Nadal, M., Schuhmacher, M., Domingo, J.L., 2018. Concentrations of trace elements and PCDD/Fs around a municipal solid waste incinerator in Girona (Catalonia, Spain). Human health risks for the population living in the neighborhood. Sci. Total Environ. 630, 34–45. https://doi.org/10.1016/j.scitotenv.2018.02.175.

[32] Bretzel, F. C., Calderisi, M., 2011. Contribution of a municipal solid waste incinerator to the trace metals in the surrounding soil. Environ. Monit. Assess. 182, 523–533. https://doi.org/10.1007/s10661-011-1894-0.

[33] Meneses, M., Llobet, J.M., Granero, S., Schuhmacher, M., Domingo, J.L., 1999. Monitoring metals in the vicinity of a municipal waste incinerator: temporal variation in soils and vegetation. Sci. Total Environ. 226, 157–164. https://doi.org/10.1016/S0048-9697(98)00386-6.

[34] Vilavert, L., Nadal, M., Schuhmacher, M., Domingo, J.L., 2015. Two decades of environmental surveillance in the vicinity of a waste incinerator: Human health risks associated with metals and PCDD/Fs. Arch. Environ. Contam. Toxicol. 69, 241–253. https://doi.org/10.1007/s00244-015-0168-1.

[35] Ma, W., Tai, L., Qiao, Z., Zhong, L., Wang, Z., Fu, K., Chen, G., 2018. Contamination source apportionment and health risk assessment of heavy metals in soil around municipal solid waste incinerator: A case study in North China. Sci. Total Environ. 631–632, 348–357. https://doi.org/10.1016/j.scitotenv.2018.03.011.

[36] Bo, X., Guo, J., Wan, R., Jia, Y., Yang, Z., Lu, Y., Wei, M., 2022. Characteristics, correlations and health risks of PCDD/Fs and heavy metals in surface soil near municipal solid waste incineration plants in Southwest China. Environ. Pollut. 298, 118816. https://doi.org/10.1016/j.envpol.2022.118816.

[37] Li, N., Kang, Y., Pan, W., Zeng, L., Zhang, Q., Luo, J., 2015. Concentration and transportation of heavy metals in vegetables and risk assessment of human exposure to bioaccessible heavy metals in soil near a waste-incinerator site, South China. Sci. Total Environ. 521–522, 144–151. https://doi.org/10.1016/j.scitotenv.2015.03.081.

[38] Li, T., Wan, Y., Ben, Y., Fan, S., Hu, J., 2017. Relative importance of different exposure routes of heavy metals for humans living near a municipal solid waste incinerator. Environ. Pollut. 226, 385–393. https://doi.org/10.1016/j.envpol.2017.04.002.

[39] Richardson, J.B., 2020. Urban forests near municipal solid waste incinerators do not show elevated trace metal or rare earth element concentrations across three cities in the northeast USA. Environ. Sci. Pollut. Res. 27, 21790–21803. https://doi.org/10.1007/s11356-020-08439-3.

[40] Li, Y., Zhang, H., Shao, L., Zhou, X., He, P., 2019a. Impact of municipal solid waste incineration on heavy metals in the surrounding soils by multivariate analysis and lead isotope analysis. J. Environ. Sci. 82, 47–56. https://doi.org/10.1016/j.jes.2019.02.020.

[41] Li, P., Wang, X., Zou, X., Yu, Z., Li, J., Yang, Y., Zhang, H., 2019b. Metallic contamination in soils around a municipal solid waste incineration site: a case study in northeast China. Environ. Sci. Pollut. Res. 26, 26339–26350. https://doi.org/10.1007/s11356-019-05763-1.
